# Origin, Spread and Demography of the *Mycobacterium tuberculosis* Complex

**DOI:** 10.1371/journal.ppat.1000160

**Published:** 2008-09-26

**Authors:** Thierry Wirth, Falk Hildebrand, Caroline Allix-Béguec, Florian Wölbeling, Tanja Kubica, Kristin Kremer, Dick van Soolingen, Sabine Rüsch-Gerdes, Camille Locht, Sylvain Brisse, Axel Meyer, Philip Supply, Stefan Niemann

**Affiliations:** 1 Lehrstuhl für Zoologie und Evolutionsbiologie, Department of Biology, University of Konstanz, Konstanz, Germany; 2 Ecole Pratique des Hautes Etudes, Muséum National d'Histoire Naturelle, UMR-CNRS 5202, Département Systématique et Evolution, Paris, France; 3 Institut Pasteur de Bruxelles, Laboratoire Tuberculose et Mycobactéries, Brussels, Belgium; 4 Research Center Borstel, Department of Clinical Medicine, Borstel, Germany; 5 National Institut of Public Health and Environment, Bilthoven, The Netherlands; 6 INSERM U629, Lille, France; 7 Institut Pasteur de Lille, Lille, France; 8 Institut Pasteur, Genotyping of Pathogens and Public Health, Paris, France; University College Cork, Ireland

## Abstract

The evolutionary timing and spread of the *Mycobacterium tuberculosis* complex (MTBC), one of the most successful groups of bacterial pathogens, remains largely unknown. Here, using mycobacterial tandem repeat sequences as genetic markers, we show that the MTBC consists of two independent clades, one composed exclusively of *M. tuberculosis* lineages from humans and the other composed of both animal and human isolates. The latter also likely derived from a human pathogenic lineage, supporting the hypothesis of an original human host. Using Bayesian statistics and experimental data on the variability of the mycobacterial markers in infected patients, we estimated the age of the MTBC at 40,000 years, coinciding with the expansion of “modern” human populations out of Africa. Furthermore, coalescence analysis revealed a strong and recent demographic expansion in almost all *M. tuberculosis* lineages, which coincides with the human population explosion over the last two centuries. These findings thus unveil the dynamic dimension of the association between human host and pathogen populations.

## Author Summary

The causative agents of tuberculosis, grouped in the *Mycobacterium tuberculosis* complex, have infected one-third of the present human population and a wide range of other mammals. However, paradigmatic questions, such as why, where and when the disease began and expanded, have largely remained unanswered. In this study, we provide genetic evidence indicating that the most common ancestor of the bacterial complex emerged some 40,000 years ago from its progenitor in East Africa, the region from where modern human populations disseminated around the same period. This initial step was followed 10,000 to 20,000 years later by the radiation of two major lineages, one of which spread from human to animals. In more recent years (approximately 180 years ago), coinciding with the human population explosion and the industrial revolution, the human-associated pathogen lineages have strongly expanded. These results thus reveal the strikingly parallel demographic evolution between humans and one of their primary pathogens.

## Introduction

The *Mycobacterium tuberculosis* complex (MTBC) is composed of closely related bacterial sub-species that have plagued human and animal populations for thousands of years. The most famous member of the MTBC is *M. tuberculosis*, the etiological agent of tuberculosis in humans that killed 1.7 million people in 2004 according to the World Health Organization [1]. A new threat is the worldwide emergence of multi-drug resistant (MDR) and extremely drug-resistant (XDR) strains. Recent data suggest that the propensity to gain drug resistance as well as the pathogen's transmissibility profile may be influenced by the genetic and evolutionary background of *M. tuberculosis* strains [2]. Thus, understanding the relationships and dynamics of the MTBC lineages will undoubtedly help to unravel the basis for the considerable success and spread of tuberculosis, in both humans and animals. The MTBC is essentially clonal with little evidence of horizontal gene exchange [3],[4],[5], and probably derived from a pool of ancestral tubercle bacilli, collectively called “*Mycobacterium prototuberculosis*” [6]. However, despite the highly successful worldwide spread of the MTBC, the evolutionary timing of this spread remains largely unknown.

This lack of knowledge is largely due to the limitations of the genetic markers used so far. All efforts to time MTBC evolution with single nucleotide polymorphisms (SNPs) have been based on a non-warranted hypothesis of universal bacterial mutation rates, itself extrapolated from a very hypothetical time of divergence between *Escherichia coli* and *Salmonella enterica* [7].

In this study, we used a completely new approach by employing genetic markers based on mycobacterial interspersed repetitive units (MIRUs) to determine the timing of divergence, population diversity and spread of the MTBC. MIRU loci comprise variable numbers of tandem repeat (VNTR) sequences, which allow them to be used as powerful genotyping markers [8],[9]. In terms of genetic diversity and mutation rates, they resemble human microsatellites, which are widely used in human population genetics studies [10]. Similar to microsatellites, MIRUs behave as selectively neutral phylogenetic markers if sufficient numbers of loci are used to buffer against potential biases.

Here we used experimental data on the variability and evolution of these markers in clinical isolates of infected patients, which allowed us to calculate the MIRU molecular clock and model their evolution in coalescence approaches. Based on this information and extensive analysis of a large collection of representative MTBC strains, we obtained new insights into the origin and demography of the MTBC and its dynamic association with the human host.

## Results

### *M. tuberculosis* phylogeny

**Figure 1:**
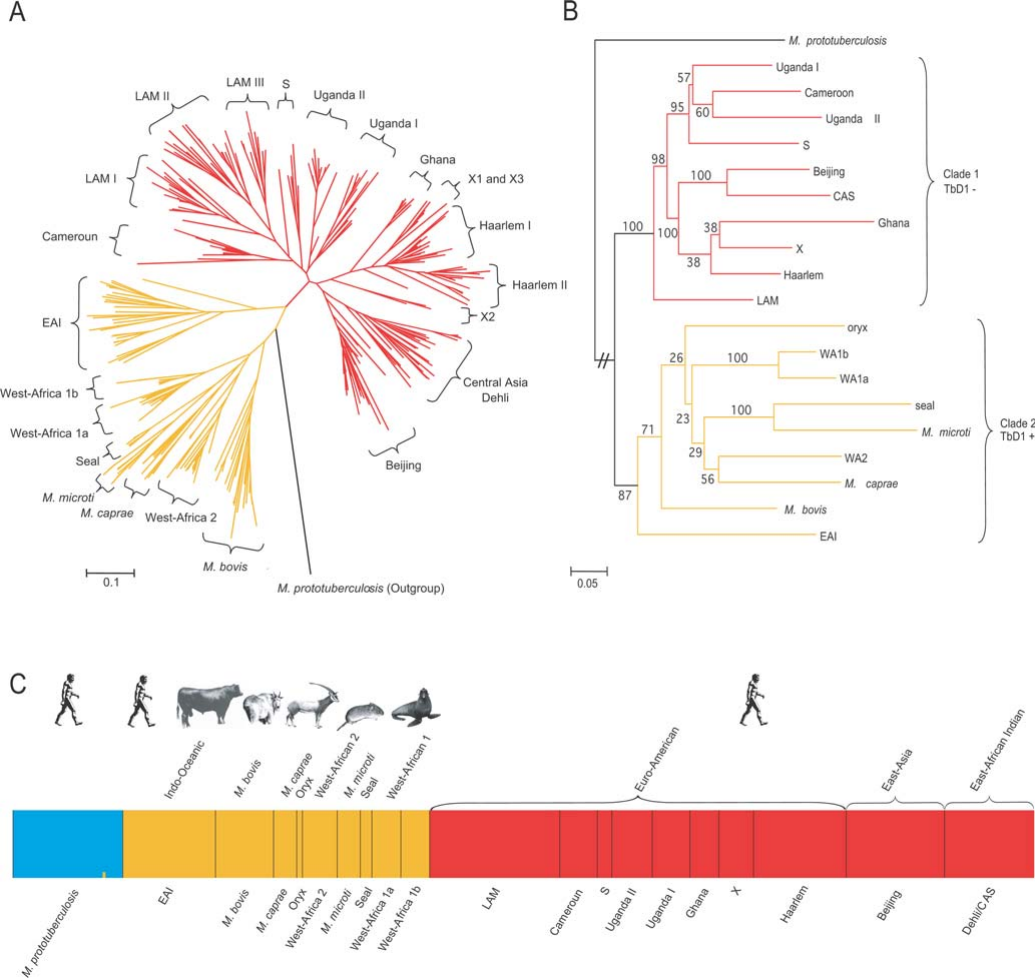
Evolutionary relationships of the *Mycobacterium tuberculosis* complex. (A) Unrooted MIRU Neighbour-joining phenogram depicting genetic distance relationships among tubercle bacilli isolates based on Nei et al.'s *D_A_* distances. (B) Rooted MIRU population Neighbour-joining tree based on genetic distance. *M. prototuberculosis* was used as an outgroup. Values on the nodes represent the percentage of bootstrap replicates over individuals (N = 1000) showing the particular nodes. Branch lengths are proportional to the genetic distance between the tubercle lineages. It is noteworthy that low bootstrap values within clade 2 prevent us from drawing further inferences on the branching order in this clade (see also main text). Wa, West-Africa. (C) Population structure of 20 MTBC clonal lineages using the no-admixture model, where *K* = 3. Each colour represents one cluster, and the length of the color segment shows the strains' estimated proportion of membership in that cluster. Results shown are averages over 10 STRUCTURE runs. For clarity, strains codes are also given according to Gagneux et al. (2006).

To infer the MTBC evolutionary history, we used a sample collection of 355 isolates, representative of well-identified primary branches of the MTBC world distribution (Table S1). A recently standardized combination of 24 MIRU loci (Figure S1), which does not comprise saturated loci [11], was utilized. To illustrate the power of MIRUs to reconstruct geographical patterns of genetic differentiation and their level of resolution, a distance-based tree was constructed using individual genotypes and a neighbour-joining algorithm (Figure 1A). The tree grouped all *M. tuberculosis sensu stricto* isolates (all from human patients) in a distinct lineage with the notable exception of the East African-Indian (EAI) population whose affiliation is unclear based on this approach. Another major lineage encompassed all MTBC strains from animals (*M. microti*. *M. bovis*, *M. caprae* and *M. pinipedii*) and the human isolates from West-Africa (*M. africanum* West African 1 and 2). From the resulting tree, it appears that the groupings of isolates within the primary MTBC branches based on SNPs, spoligotyping and large sequence polymorphisms (LSPs) [12],[13],[14],[15],[16],[17] (Figure S2) are highly congruent with those based on the MIRU typing, albeit the branch resolution was higher in the latter. In order to more robustly define the relationships between the lineages (by reducing the number of individuals vs the number of markers), we then grouped individual isolates into the populations defined by the above groupings and built a tree based on MIRU allelic frequencies in these populations (Figure 1B). The tree was rooted with samples of *M. prototuberculosis* (including *M. canettii*), which was recently reported to represent the progenitor of the MTBC [6]. This approach clearly revealed the distinctiveness of the two major lineages with strong bootstrap support, called hereafter clades 1 and 2. A further geographic sub-structuring within clade 1 became apparent, with distinct branches for the African (Uganda, Cameroon and S), Asian (Beijing and CAS), Latin American-Mediterranean and African-European populations (X, Ghana and Haarlem). Clade 2 is composed of both animal and human pathogenic isolates. A basal position of EAI (human tuberculosis) in clade 2 has strong statistical support, indicating a human origin for this predominantly animal-associated MTBC lineage. However, low bootstrap values within clade 2 prevent us from drawing further inferences on the branching order.

### A population genetics perspective

To confirm the groupings and the deep dichotomy obtained with the MIRUs, we used an independent approach, based on the ‘no-admixture’ model of the STRUCTURE program [18]. In this Bayesian approach, multilocus genotypic data are used to define a set of populations with distinct allele frequencies and assign individuals probabilistically to them, with or without prior knowledge of geographic sampling information. We applied STRUCTURE to the global data set (including the outgroup) and in ten independent runs, at K = 3 populations (Fig. 1C) STRUCTURE detected the same two deeply divergent clades 1 and 2 that were identified with the neighbour joining analysis (see Figure 1B). Notably, this separation is independently supported by the fact that TbD1 (*M. tuberculosis* deletion 1) is lacking in all clade 1 strains but present in all clade 2 strains, including those from EAI (Figure 1B and S2) [12]. The robustness of these clades was further evidenced by STRUCTURE analysis, because each isolate derived all of its MIRU's from only one of the three ancestral sources of clade 1, clade 2 or *M. prototuberculosis* (see Protocol S1). We further modelled the Bayesian assignments of the two main clades by sub-dividing them into additional clusters (Figure S3A). The bacterial isolates were consistently split into the same major clusters as those defined by the distance-based approach (see above). The highest likelihoods were obtained for *K* = 6 populations in each of the two main clades. Only three isolates (0.85%) were assigned to unexpected clusters by the Bayesian approach (Figure S3A), further illustrating the consistency of MIRU-VNTR cluster designations. To detect possible horizontal genetic transfer events, we used the STRUCTURE ‘linkage model’ as was done to detect ongoing genetic exchange in *Helicobacter pylori* [19],[20], *Escherichia coli* [21] and *Moraxella catarrhalis* [22]. Runs without prior knowledge of population source (Figure S3B) suggested that the vast majority of the MTBC strains are clonal, while some *M. prototuberculosis* strains might be hybrids with MTBC genotypes, in accordance with previous results [3],[5],[6].

### MTBC ancestral lineages and genetic diversity

**Figure 2:**
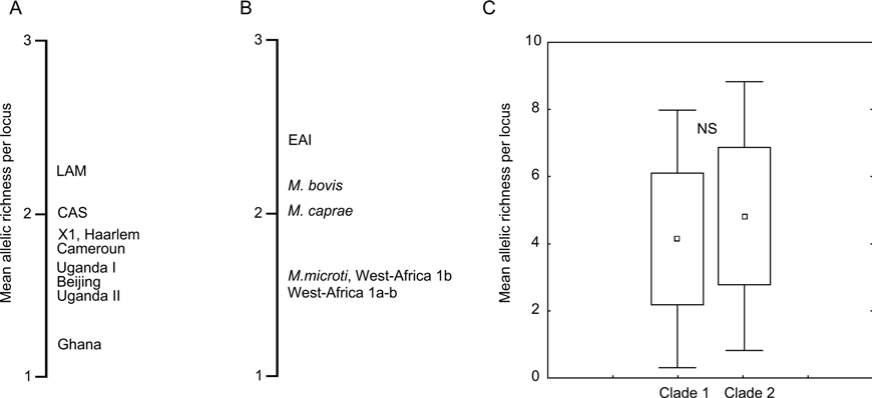
Genetic variability in the different MTBC lineages. (A and B) MIRU allelic richness in each population within clade 1 and 2 respectively. Rarefaction included eight isolates per population (smaller populations were not considered in this analysis. (C) Clades mean allelic richness. Notice that the difference between clade 1 and 2 is not significant (t-test, *P* = 0.08).

To further assess the deep dichotomy, we calculated the allelic richness (the number of alleles) of the populations within the two main clades after correcting for sample size effects [23] (Fig. 2). High levels of genetic diversity are a surrogate indication of ancestral origins as illustrated in the highly divergent African human populations. The mean allelic richness per locus was close to five for both clades, and the difference was not significant (Fig. 2C), arguing for a simultaneous split of the two clades. As expected, LAM and EAI, the most basal populations in clades 1 and 2 respectively, contained the highest number of alleles (Fig. 2A, 2B). However, some uncertainty remains on a basal position for LAM because it conflicts with groupings based on internal deletions of the pks15/1 gene and on SNPs [13].

### Dating the disease and the evolutionary radiation steps

In order to estimate the time to the most recent common ancestor (TMRCA) in the MTBC, we made use of recent analytical tools [24],[25], which make these estimations possible. They rely on Bayesian statistics and apply a stepwise mutation model (SMM) for genetic markers. This model is a reasonable assumption for MIRU mutations, as initially shown for MIRU locus 4 in the BCG evolutionary framework [9]. To test the validity of this model for the total set of the MIRU loci used, we built a minimal spanning tree of all MTBC strains based on the degree of allele sharing. We then evaluated the proportion of strains that differed from their closest relative by one step (single-locus variants- SLVs) or by multiple steps, which would violate the SMM model. This simple method will certainly overestimate any violations of the SMM model because our sampling scheme is not exhaustive, resulting in some spurious missing links (intermediate strains) that falsely invalidate the SMM model. However, the data showed that at least 64% of the allelic changes fit the stepwise mutation model, a result that is close to the 75% and 81% observed in *E. coli* and yeast VNTRs, respectively [26],[27].

To further evaluate the validity of the SMM model, eBURST analysis was performed on a much larger dataset comprising 1,733 MIRU-VNTR profiles from two population-based studies performed at regional and national levels (see Materials and Methods). This analysis identified 142 groups and 1061 singletons. In order to determine whether tandem repeats evolve following a SMM model and to detect a potential bias towards increase or decrease in repeat numbers, we computed within each eBURST group all differences in number of repeats along the evolutionary path, starting from the putative founder of the group to its surrounding SLVs (Figure S4). For all but two of the 24 loci, the most frequent change was either −1 or +1 repeat unit, with the symmetric change generally being the next most frequent. The only minor exceptions were loci MIRU-VNTR 3007 and 2347, which contain little information, because the only changes were one occurrence each of −2 and +1 repeat units, and four occurrences of −2 and two of +1 respectively. Both for the individual loci and for data cumulated over the 24 loci (Figure S5), the distribution of occurrences was unimodal and centered on 0 (average of −0.07±0.23, CI = 0.95, for cumulated data). At least sixty-five percent of the allelic changes matched the stepwise mutation model. It is noteworthy that missing links falsely invalidating the SMM model probably occur even in this population-based dataset, because many patients from the population studied (from the Brussels region and the entire Netherlands) were foreign-born and have probably acquired their infection abroad. Therefore, tandem repeats in *M. tuberculosis* most frequently evolve by progressive gain or loss of single repeat units without significant general bias towards increase or decrease.

**Figure 3:**
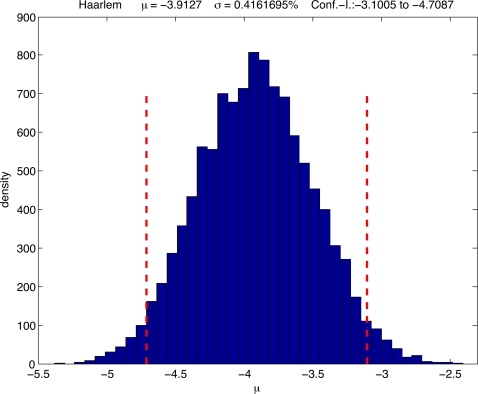
Calculated posterior mean for MIRU-VNTR mutation rate among loci using the MSVAR algorithm. This graph corresponds to the output obtained for the Haarlem population sample and the 95% interval confidence is given (red dotted lines).

To estimate MIRU mutation rates, we used data from large sets of serial or epidemiologically-linked isolates. The probability of showing a repeat change over periods of up to 7 years was estimated to be about 1% for five of the most variable loci [11]. This corresponds to a single-locus mutation rate of 1.4×10^−3^ per year. Consistently, 4 of these 5 loci composed the top 4 in the hierarchy of single-locus variation frequencies measured among the MIRU loci, both in a global MTBC isolate dataset [11] and in the above population-based dataset (data not shown). This supports the use of these frequencies as a surrogate for estimating relative mutation rates of the different markers, and especially those of the less variable loci, for which repeat changes among serial or epidemiologically-linked isolates were not observed [11]. We therefore somewhat arbitrarily chose a lower mean mutation rate per year of 10^−4^ as a prior for the Bayesian inferences [25] over all loci, in order to accommodate the less variable loci which were associated with up to 38fold lower frequencies of single-locus variation. It is noteworthy that this initial value was well supported by posterior Bayesian analysis, as the calculated posterior mean for the mutation rate was 10^−3.91^ (Figure 3). By applying this mutation rate and a generation time of one day for the tuberculosis bacilli, we estimated a mean TMRCA of ≈40,000 years before present for the complex (Table 1). The TMRCAs for clades 1 and 2 were estimated as 21,000 and 33,000 years, respectively, and two of the oldest lineages, EAI and LAM coalesced at 13,700 and 7,000 years, respectively (Table 1).

**Table 1:** Estimated Times (in years) since the most recent common ancestor (TMRCA).

TMRCA	Age in years	CIs	Hierarchic level
LAM-Beijing	21,300	(14,300–31,600)	Clade 1
Beijing-CAS	17,100	(11,600–25,400)	Asian TB
LAM-LAM	7,060	(4,370–11,100)	LAM
CAS-CAS	9,450	(6,100–14,700)	CAS
EAI-WA2	32,800	(27,900–38,300)	Clade2
EAI-EAI	13,700	(9,100–21,000)	EAI
*M. bovis*-*M. bovis*	5,750	(4,560–7230)	*M. bovis*
EAI-LAM	41,500	(29,100–60,000)	MTBC
EAI-Beijing	37,500	(25,800–55,100)	MTBC

Estimates and 95% confidence intervals were calculated with the software YTime.

**Figure 4:**
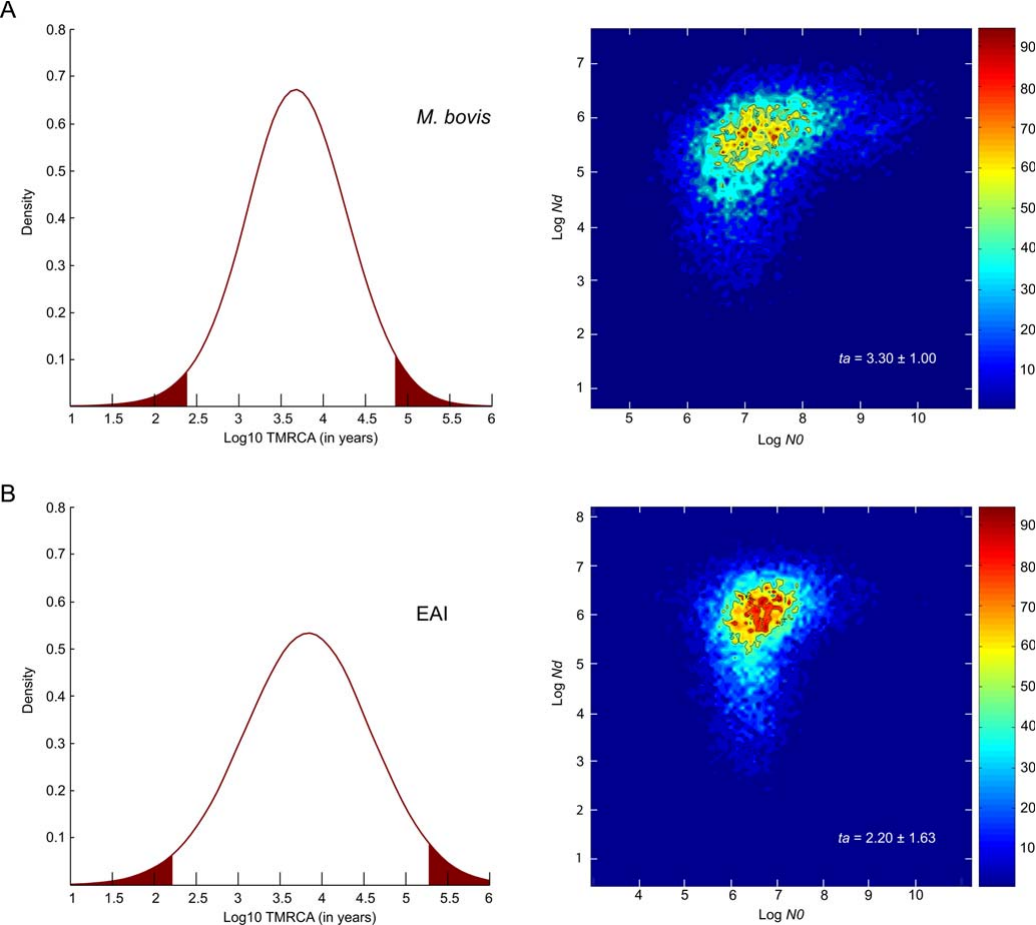
Detection of recent expansion in different MTBC lineages. (A) Posterior distribution of *M. bovis* TMRCA, including the 95% confidence interval and density plots of the marginal posterior distribution of log (*N_0_*), where *N_0_* is the current effective number of chromosomes and log (*N_d_*), where *N_d_* is the number of chromosomes before expansion. (B) Same plots for EAI. *t_a_* is expressed in years (±SD) and denotes the time that has elapsed since the population growth began.

In a second step, we used the MSVAR software [25] that infers past demographic changes and calculates additional parameters, including TMRCA of monophyletic populations using slightly different algorithms. For this procedure, we focused on lineages for which at least 30 isolates were included in the study, in order to avoid small sample size artefacts. The use of this method confirmed the TMRCA of the EAI population at ≈7,000 years (Figure 4B and Table 2), albeit with very wide confidence intervals (150–190,000 years).

**Table 2:** Time to the most recent common ancestor (TMRCA), time elapsed since the last expansion began (*t_a_*) and growth rate estimates based on the MSVAR software.

	TMRCA			*t_a_*			Growth
	lower	modal	upper	lower	modal	upper	modal
Africa	2.024	3.510	5.085	0.418	2.193	4.604	0.60
Asia	2.126	3.620	5.022	0.833	2.006	3.774	2.16
Europe	2.257	3.701	5.029	0.710	2.321	3.527	2.12
Beijing	0.939	3.040	5.396	0.566	2.514	4.421	2.71
CAS	1.865	3.540	5.156	0.389	2.345	3.624	1.67
LAM	2.378	4.007	5.758	1.341	2.989	4.381	1.80
EAI	2.208	3.854	5.282	0.134	2.145	6.274	0.81
*M. bovis*	2.379	3.687	4.859	1.316	3.184	5.222	1.83

Modal values and 95% confidence intervals are presented. The results are on a log scale.

### *M. tuberculosis* demographic expansion

Finally, genetic data can also unravel recent demographic change signatures in bacterial populations. By using Bayesian statistics, we tested whether a recent decline or expansion occurred in the MTBC population, and calculated *t_a_*, which reflects the time that has elapsed since the decline or expansion began. All MTBC populations from human sources that we considered displayed markedly consistent expansion rates and EAI is typical in that respect (see Figure 3B). The detected growth rates (on a log scale) ranged from a modest 0.6 value, as seen in Africa, to 2.7 for Beijing, which is probably the most successful present day lineage. This latter value translates into a recent 500-fold population size increase. The mean modal value of log10 *t_a_* was 2.25 (range 2.00–2.5) for the different populations, with the exception of the LAM lineage. This corresponds to a tuberculosis expansion that began 180 years ago (see Table 2).

## Discussion

**Figure 5:**
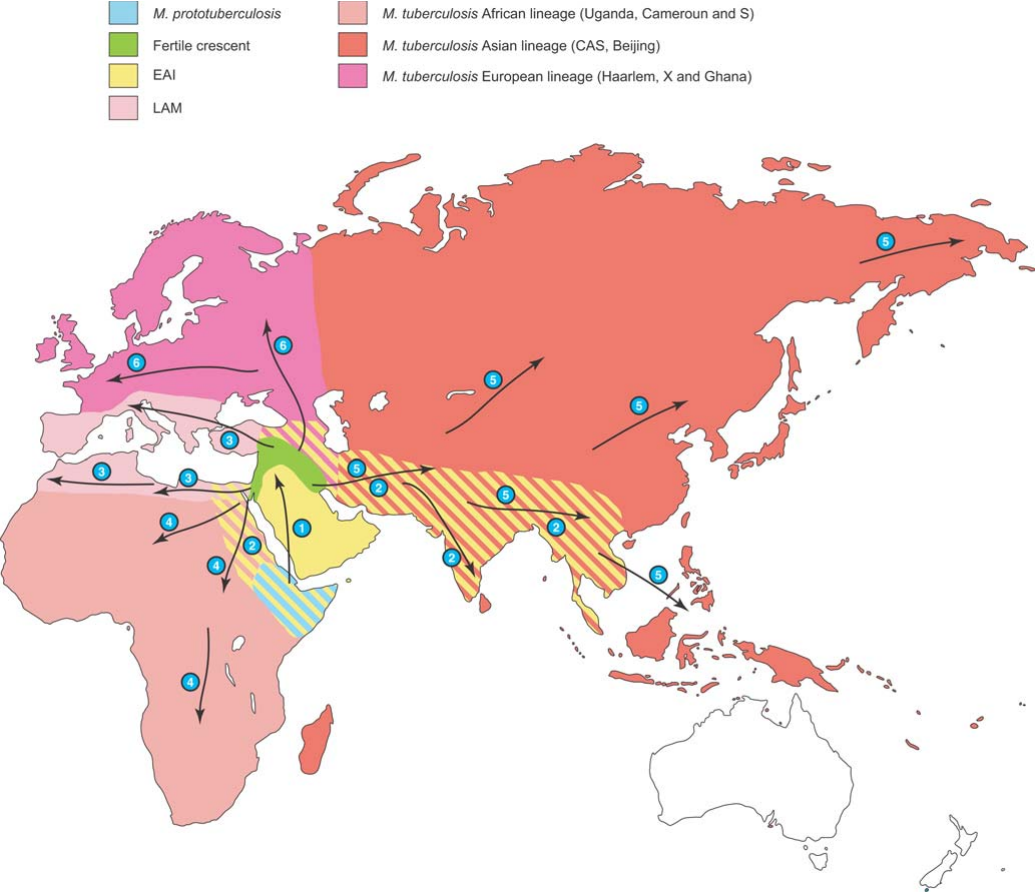
*M. tuberculosis* evolutionary scenario (out of Mesopotamia). The main migrations events are numbered and correspond to: 1, *M. prototuberculosis*, the ancestor of the MTBC, this bacterium reached the Fertile Crescent some 40,000 years ago by sea or land; 2 and 3, two distinct basal lineages arose, EAI and LAM and spread out of Mesopotamia some 10, 000 years ago; 4, 5 and 6, later on (8–5000 years ago) derived populations from clade 1 followed main human migration patterns to Africa, Asia and Europe, giving rise to locally adapted tubercle strains and further diversifications. Note that the depicted borders are “artificial” and are used for the demonstration. Global movements and intercontinental exchanges tend to blur this phylogenetic signal though strong enough to be detected nowadays.

Taken together, the findings presented in this study indicate that the MTBC is composed of two major lineages and has emerged approximately 40,000 years ago. This estimate is strikingly close to the proposed time of dispersal of founder modern human populations from the Horn of Africa [28]. However this dating must be considered with caution in the light of the large confidence intervals. Our results support the emergence of the MTBC clone from the *M. prototuberculosis* progenitor pool and its co-migration with modern humans out of Africa [6]. A similar trend was recently proposed for *H. pylori* and *M. leprae* [29],[30]. We suggest that two main lineages arose later some 20,000 to 30,000 years ago from the common MTBC ancestor, one of which spread exclusively among humans, with subsequent waves of migration to Asia, Europe and continental Africa (Figure 5). This spreading scenario fits well with the current worldwide distribution of the main MTBC lineages, as reflected by the SpolDB4 database [12],[13],[14],[15],[16],[17] and LSP analysis [14],[17]. The second lineage (clade 2) arose from a human EAI-like population some 30,000 years ago and is the probable source of animal tuberculosis [12],[31], a derivation that is strongly and convergently supported by both distance-based and probabilistic methods (i.e. NJ and STRUCTURE). This conclusion is consistent with the finding that extant representatives of *M. tuberculosis*, which derived from the proposed progenitor of MTBC, are human pathogens [6]. Thus it is likely that humans infected their livestock and not the other way around. Clade 2 secondary branches include *M. bovis* and *M. caprae*, the infectious agents of tuberculosis in a wide variety of animals including cattle and goat, which were first domesticated in the Near East [32],[33]. The transition from human to animal hosts may thus be linked to plant and animal domestication that took place in the Fertile Crescent some 13,000 years ago. This period corresponds to the estimated time of diversification of the oldest EAI and LAM populations (Table 2). In the Fertile Crescent, and during that era of human history, small nomadic hunter-gatherer groups were replaced by farming societies based on domesticated livestock and crops [34]. This paramount event in human history was probably not without consequence for an epidemic, infectious disease such as tuberculosis, where crowded farming populations may have promoted high infection rates, bacterial spread and transition to new niches and animal hosts [35]. Clade 2 also includes *M. africanum* strains that primarily infect humans. However, it has recently been speculated that *M. africanum* may not be primarily adapted to the human host but might have originated from an unknown animal reservoir [36].

All MTBC populations from human sources displayed markedly constant expansion rates, corresponding to an expansion that dates back to only about 180 years. Furthermore, the largest population size increase (500-fold) was detected for Beijing, which is thought to be the most successful present day lineage. These results suggest that the expansion of the most recent form of human tuberculosis was coupled to Western urbanization and industrialization. This expansion was synchronous with the modern demographic explosion of *Homo sapiens* and modern intercontinental movements. Evidence for strong phylogeographical structuring of the pathogen population and preferential sympatric combinations of pathogen populations with particular ethnic groups has indicated a close association between *M. tuberculosis* and its human host [3],[13],[37]. Our results indicate recent parallel demographic changes between the pathogen and its host and reveal the tell-tale dynamic dimension of this association. The coalescence approach may also be useful in the future to monitor demographic changes in emerging MDR *M. tuberculosis* strains.

Some of the conclusions presented here on the basis of MIRU data have also been reached previously, e.g. data from comparative genomics [12] after the completion of *M. bovis* genome [31] indicated that the MTBC did not arise as a zoonosis [38]. In contrast, the validity of efforts to date the origins of the common ancestor of MTBC by using SNP-based methods [39],[40],[41], has remained questionable [42]. Furthermore, preliminary SNP-based phylogenetic reconstructions may have been affected by hitch-hiking, and ascertainment bias [43], because those SNPs were associated with genes involved in drug-resistance [44] or were selected from a non-representative set of available genomes [14],[17],[45]. Such markers evolve too slowly for recent pathogens, as is also the case for LSPs and their use often results in uninformative phylogenies that consist of multifurcated unresolved trees [13],[44]. Unlike previous studies, the novel analyses presented here rely on globally neutral markers with mutation rates that have been estimated from human *M. tuberculosis* infection cases, a descent-sampling scheme and multiple, convergent population genetic estimators. As they are based on intrinsically rare and stochastic VNTR changes in clonal populations, our mutation rate estimates do involve some special assumptions. The accuracy of the demographic and temporal estimates could be improved with long-term analyses, and we are aware that the use of a mean mutation rate for all loci is suboptimal, leading to an increase of the variance of parameters. However, our estimates were consistently corroborated by posterior Bayesian calculations in independent runs over different strain populations (ranging from 10^−4.19^ for LAM to 10^−3.82^ for EAI), ruling out the risk of some local maxima. To gain further insights into the host-pathogen interactions, it would certainly be important to account for the biogeographic history and distribution of the different *M. tuberculosis* lineages, because recent adaptations to local host populations might play a major role [13]. Furthermore, it is known, that genetic diversity can influence the transmission dynamics of drug-resistant bacteria [2],[46], and, in terms of vaccination, it would be advisable to scrutinize independently the highly polymorphic clade 2 EAI strains that markedly differ in their genetic structure from the other human tuberculosis strains.

## Materials and Methods

### Sampling and data collection

The 355 *M. tuberculosis* and *M. prototuberculosis* isolates were genotyped by multiplex PCR amplification as described previously [8],[47]. The samples were subjected to electrophoresis using ABI 3100 and 3730 automated sequencers. Sizing of the PCR fragments and assignment of the VNTR alleles of the 24 loci was done using the GeneScan and customized Genotyper, as well as the GeneMapper software packages (PE Applied Biosystems).

### Genetic diversity estimation

The number of alleles (allelic richness) in each *M. tuberculosis* complex population was estimated and sample sizes were corrected by the rarefaction procedure using HP-RARE [23]. Comparison tests as well as *P*-values were estimated using the STATISTICA v.6.1 package.

### Phylogenetic inferences

Nei et al.'s *D_A_* distance [48] was used to construct both isolate and population trees using a neighbour-joining algorithm as implemented in the software Populations version1.2.28. Support for the tree nodes was assessed by bootstrapping over loci (1, 000 iterations).

### Inferring population structure and recombination in the *M. tuberculosis* complex

Using the no-admixture model [18] (STRUCTURE version 2), three to ten parallel Markov chains were run for all models of K with a burn-in of 100,000 iterations and a run length of 10^6^ iterations following the burn-in. For each run, the ln likelihood of each model was calculated. The full data set was analysed for all models from K = 1 through to 3 without specifying prior information concerning the geographical sources or former designations. For K = 3, a clear splitting solution was found in which the sampled populations clustered into two main tuberculosis groups plus the outgroup (*M. prototuberculosis*); a result fully consistent with the neighbour-joining population tree (Figure 1B). For further analysis the data set was sub-divided into clades 1 and 2, and these were subsequently tested for K = 1 through to 6. Using the linkage model [49] of STRUCTURE version 2, ten parallel Markov chains were run for each model with a burn-in of 100,000 iterations and a run length of 10^6^ iterations following the burn-in. For each run, *M. tuberculosis* strains were specified as belonging to pre-determined source clusters. We estimated the ancestry in each source cluster and the proportion of each strain genome having ancestry in each cluster.

### Stepwise mutation model (SMM) and mutation rate estimates

To estimate the validity of SMM model, we built a minimal spanning tree of all MTBC strains based on the degree of allele sharing, by using BIONUMERICS (Applied Maths, Belgium). We then evaluated the proportion of single-locus variants (i.e. strains that differed from their closest relative) that differed by one or by multiple repeat-changes. To further evaluate the validity of the SMM model and to detect a potential bias towards increase or decrease in repeat numbers, eBURST analysis was performed on a larger dataset from two population-based studies. The first one included 807 isolates from different TB cases notified in the Brussels-Capital Region (Belgium) from September 1^st^, 2002 to December 31^st^, 2005 [50], while the second one is an ongoing study including 1907 isolates from different TB cases notified in the Netherlands over 2004 and 2005 (Van Soolingen et al., unpublished). In total, the dataset included 1,733 MIRU-VNTR profiles, with no missing data or incomplete repeats. On this dataset, the differences in the number of repeats were calculated for each pair of ancestor/descendant genotypes along the evolutionary path inferred by eBURST analysis [51]. The occurrence of each value of repeat difference was recorded for each group (defined as groups of strains with at most one allelic mismatch with at least one other member of the group), and values were pooled over all eBURST groups. This analysis was performed using software Multilocus Analyzer (S. Brisse, unpublished), which is an independent implementation (coded in Python) of the eBURST algorithm, to which the SMM test function was added.

MIRU mutation rates were estimated by using data on VNTR changes among large sets of serial or epidemiologically-linked isolates [11]. Single-locus mutation rates of 5 most variable loci were estimated from corresponding frequencies of observed repeat changes. Repeat changes among serial or epidemiologically-linked isolates were not detected among the remaining, less variable loci. Therefore, the relative frequencies of single-locus variations among closely related isolates in a global MTBC isolate dataset [11] and in the population based dataset (see above) were then used as a surrogate for estimating mutation rates of less variable markers relatively to these most variable loci.

### Coalescence, TMRCA and demography

In a first step, we used a Bayesian approach [25] that assumes a stepwise mutation model and estimates the posterior probability distributions of the genealogical and demographic parameters of a sample using Markov chain Monte Carlo simulations based on MIRU data. This method permits to extrapolate important biological parameters like the TMRCA of a given sample in years, the past and present effective population size and the latest demographic changes (decline, constant population size or expansion). In order to assess the age of the main *M. tuberculosis* lineages, an alternative algorithm, YTime [24] was used to calculate the TMRCAs and their confidence intervals. For the MSVAR procedure [25],[52], we focused on lineages of which at least 30 isolates were available, in order to obtain a reliable coverage of the TMRCA and to avoid small sample size artefacts. The estimated parameters were scaled in terms of current population size, and two main demographic parameters were quantified: *t_f_*, which is a measure of time in generations, was defined as *t_a_*/*N_0_*, where *t_a_* denotes the number of generations that have elapsed since the decline or expansion began, and *r*, which was defined as *N_0_*/*N_1_*, where *N_0_* is the current effective number of chromosomes, and *N_1_* is the number of chromosomes at some previous point in time *t_f_*. For a declining population *r*<1, for a stable population *r* = 1 and for expanding populations *r*>1. The procedure also estimates *θ*, which is defined as *N_0_μ*, where *μ* is the mutation rate (mutation locus^−1^ generation^−1^). The analyses were performed assuming exponential demographic change. Three different chains were run for each analysis to confirm the convergence of the results. In the analyses, rectangular priors of the log parameter values have been used. The method was found to converge appropriately for both single and multilocus data sets and supported a model of population expansion for all MTBC populations. We present only the multilocus data in the present report. Expansion signatures were robust and were confirmed in runs where decline was assumed as a prior (10^−2^–10^−3^).

YTime [24]: YTime is a Matlab function which calculates the TMRCA for haplotype linked loci under the assumption of an S-SSM, which allows for unbiased +/−1 steps. YTime calculates confidence intervals using a simulation approach and is independent of the shape of the genealogy. We used all available loci (N = 24) as an input. The strains were grouped according to their lineages (obtained by phylogenetic analyses). The ancestral genotype for every subgroup was calculated as the mean of every single locus in the particular subgroup. The mutation rate was 10^−4^ per year per locus. For the growth rate parameters we assumed a mean effective population size of 10^8^ for every sub-population and a growth of 10^3^ (the mean of the results is not affected by the growth rate, just the confidence intervals).

## Supporting Information

Protocol S1 (0.06 MB DOC)

Table S1 List of the MTBC isolates used in this study.
(0.08 MB DOC)

Figure S1 Bubble-graph representation of allele frequencies for the different MIRU loci. Allele size (number of repeats) on the y-axis, and source populations on the x-axis.
(2.07 MB PDF)

Figure S2 MIRU and region of deletion (RD) patterns of 176 random selected *M. tuberculosis* and *M. prototuberculosis* strains. A visualisation of MIRU and RD data was added to the rooted population neighbour-joining tree based on genetic distances (see Figure 1B). Representative results are shown for 89 isolates. The copy numbers of the 24 MIRU loci are displayed in blue shades ranging from 0 (white) to 13 (dark blue). For RD-analysis, black and white boxes correspond respectively to presence and absence of the considered region. The deletions distribution and the spolygotype patterns (data not shown) were in good congruence with the MIRU typing. Several clusters defined by MIRU typing also showed specific deletions such as RD726 for the Cameroon lineage or RD711 for West-African 1 strains. The presence or absence of the deletions also supported the dichotomy of the tree as all clade 1 strains are TBD1 negative and all clade 2 strains are TBD1 positive. However, it must be noted, that MIRU typing allowed a fin grain resolution, for example, several lineages e.g. West African 1a and West African 1b belong to two different lineages but remain undistinguishable by RD-typing. The presence or absence of the 16 Rds was determined by PCR as described previously [15],[16],[17],[18].
(1.21 MB EPS)

Figure S3 MTBC population structure. (A) Population structure of 355 *M. tuberculosis* and *M. prototuberculosis* isolates. Each strain is represented by a single vertical line divided into *K* colours, where *K* is the number of clusters assumed. Each colour represents one cluster, and the length of the coloured segment shows the strain's estimated proportion of membership in that cluster. Black lines separate the main lineages. (B) Population structure for *K* = 3, as in a, but with the implementation of the linkage model.
(7.91 MB EPS)

Figure S4 Evolution of repeat copy number among MIRU-VNTR single-locus variants. To evaluate the validity of the stepwise mutation model and to detect a potential bias towards increase or decrease in repeat numbers, EBURST analysis was performed on a large dataset comprising a total of 2714 isolates from two population-based studies. Genotypes from a selected clonal complex are represented as circles. Stepwise and non-stepwise allelic changes between genotypes, along with corresponding marker number, are highlighted in green and gray, respectively. Insets show examples of allelic identification by analysis of marker amplicons using GENEMAPPER. Gray ladders and axis in insets define amplicon size bins expected for MIRU-VNTR alleles and measured amplicon sizes in base pairs, respectively. Code numbers in the upper left of insets define sample and marker identity, respectively. M, marker (from 1 to 24).
(2.07 MB EPS)

Figure S5 Distribution of repeat copy number changes among MIRU-VNTR single-locus variants. The difference in the number of repeats was calculated for each pair of ancestor/descendant genotypes along the evolutionary path inferred by EBURST analysis, on a large dataset comprising a total of 2714 isolates from two population-based studies. The occurrence and nature of each repeat difference was recorded for each strain group (defined as groups of strains with at most one allelic mismatch with at least one other member of the group), and values were pooled over all EBURST groups.
(0.96 MB EPS)
