# Supplementary material for: Origin, Spread and Demography of the Mycobacterium tuberculosis Complex
Source: PLoS Pathog. 2008 Sep 26;4(9):e1000160. doi: 10.1371/journal.ppat.1000160 (PMC2528947; doi:10.1371/journal.ppat.1000160)
Supplement: Protocol S1 — (0.06 MB DOC) [file ppat.1000160.s001.doc]

# Methods

# Sampling and data collection

The 355 *M. tuberculosis* and *M. prototuberculosis* isolates were genotyped by multiplex PCR amplification as described previously [1,2]. The samples were subjected to electrophoresis using ABI 3100 and 3730 automated sequencers. Sizing of the PCR fragments and assignment of the VNTR alleles of the 24 loci was done using the Genscan and customized Genotyper, as well as the GeneMapper software packages (PE Applied Biosystems).

## Genetic diversity estimation

The number of alleles (allelic richness) in each *M. tuberculosis* complex population was estimated and sample sizes were corrected by the rarefaction procedure using HP-Rare [3]. Comparison tests as well as *P*-values were estimated using the Statistica v.6.1 package.

# Phylogenetic inferences

Nei et al.’s *D_A_* distance [4] was used to construct both isolate and population trees using a neighbour-joining algorithm as implemented in the software Populations version1.2.28. Support for the tree nodes was assessed by bootstrapping over loci (1, 000 iterations).

**Inferring population structure and recombination in the *M. tuberculosis* complex**

Using the no-admixture model [5] (Structure version 2), three to ten parallel Markov chains were run for all models of K with a burn-in of 100,000 iterations and a run length of 10^6^ iterations following the burn-in. For each run, the ln likelihood of each model was calculated. The full data set was analysed for all models from K = 1 through to 3 without specifying prior information concerning the geographical sources or former designations. For K = 3, a clear splitting solution was found in which the sampled populations clustered into two main TB groups plus the outgroup (*M. prototuberculosis*); a result fully consistent with the neighbour-joining population tree (Figure 1B). For further analysis the data set was sub-divided into clades 1 and 2 and these were subsequently tested for K = 1 through to 6. Using the linkage model [6] of Structure version 2, ten parallel Markov chains were run for each model with a burn-in of 100,000 iterations and a run length of 10^6^ iterations following the burn-in. For each run, *M. tuberculosis* strains were specified as belonging to pre-determined source clusters. We estimated the ancestry in each source cluster and the proportion of each strain genome having ancestry in each cluster.

### Stepwise mutation model (SMM) and mutation rate estimates

All the coalescent models are based on a simple stepwise mutation model (S-SMM). It is therefore important to check if the MIRUs loci used in this study fit this expectation as locus 4 MIRU does in the BCG evolutionary framework [7]. For this purpose, we built a minimal spanning tree (MS_TREE_) of all MTBC strains based on the degree of allele sharing. We then evaluated the proportion of strains that differed from their closest relative by one or by multiple steps. This method is a proxy that certainly overestimates the violation of the SMM model. Our sampling scheme is not exhaustive; therefore some missing links (intermediate strains) will occur and falsely invalidate the S-SMM model. However, we were able to show that at least 64% to 65% of the allelic changes fit the stepwise mutation model, a result that is close to the 75 % observed in *E. coli* variable-number tandem repeats [10] (VNTR) and the 81% in yeast [11].

# Coalescence, TMRCA and demography

In a first step, we used a Bayesian approach [12] that assumes a stepwise mutation model and estimates the posterior probability distributions of the genealogical and demographic parameters of a sample using Markov chain Monte Carlo simulations based on MIRU data. This method permits to extrapolate important biological parameters like the TMRCA of a given sample in years, the past and present effective population size and the latest demographic changes (decline, constant population size or expansion). In order to assess the age of the main *M. tuberculosis* lineages, an alternative algorithm, Ytime [13] was used to calculate the TMRCAs and their confidence intervals (for details see supporting online material). MsVar [12,14]: For this procedure, we focused on lineages of which at least 30 isolates were available, in order to get a reliable cover of the TMRCA and to avoid small sample size artefacts. The estimated parameters are scaled in terms of current population size and two main demographic parameters are quantified: *t_f_* , which is a measure of time in generations, is defined as *t_a_*/*N_0_*, where *t_a_* denotes the number of generations that have elapsed since the decline or expansion began, and *r*, which is defined as *N_0_*/*N_1_*, where *N_0_* is the current effective number of chromosomes and *N_1_* is the number of chromosomes at some previous point in time *t_f_*. For a declining population *r* < 1, for a stable population *r* = 1 and for expanding populations *r* > 1. The procedure also estimates *θ*, which is defined as *N_0_μ*, where *μ* is the mutation rate (mutation locus^-1^ generation^–1^). The analyses were performed assuming exponential demographic change. Three different chains were run for each analysis to confirm the convergence of the results. In the analyses, rectangular priors of the log parameter values have been used. The method was found to converge appropriately for both single and multilocus data sets and supported a model of population expansion for all TB complex populations. We present only the multilocus data in the present report. Expansion signatures were robust and were confirmed in runs where decline was assumed as a prior (10^-2^ – 10^-3^).

Ytime [13]: Ytime is a Matlab function which calculates the TMRCA for haplotype linked loci under the assumption of an S-SSM (Simple Stepwise Mutation Model), which allows for unbiased + /- 1 steps. Ytime calculates confidence intervals using a simulation approach and is independent of the shape of the genealogy. We used all available loci (N = 24) as an input. The strains were grouped according to their lineages (obtained by phylogenetic analyses and lab routine). The ancestral genotype for every subgroup was calculated as the mean of every single locus in the particular subgroup. The mutation rate was 10^-4^ per year per locus. For the growth rate parameters we assumed a mean effective population size of 10^8^ for every sub-population and a growth of 10^3^ (the mean of the results is not affected by the growth rate, just the confidence intervals).

References

1. Supply P, Lesjean S, Savine E, Kremer K, van Soolingen D, et al. (2001) Automated high-throughput genotyping for study of global epidemiology of *Mycobacterium tuberculosis* based on mycobacterial interspersed repetitive units. J Clin Microbiol 39: 3563-3571.

2. Supply P, Allix C, Lesjean S, Cardoso-Oelemann M, Rusch-Gerdes S, et al. (2006) Proposal for standardization of optimized Mycobacterial Interspersed Repetitive Unit-Variable Number Tandem Repeat typing of Mycobacterium tuberculosis. J Clin Microbiol.

3. Kalinowski ST (2005) HP-rare: a computer program for performing rarefaction on measures of allelic diversity. Molecular Ecology Notes 5: 187-189.

4. Nei M, Tajima F, Tateno Y (1983) Accuracy of estimated phylogenetic trees from molecular data. II. Gene frequency data. J Mol Evol 19: 153-170.

5. Pritchard JK, Stephens M, Donnelly P (2000) Inference of population structure using multilocus genotype data. Genetics 155: 945-959.

6. Falush D, Stephens M, Pritchard JK (2003) Inference of population structure using multilocus genotype data: linked loci and correlated allele frequencies. Genetics 164: 1567-1587.

7. Supply P, Mazars E, Lesjean S, Vincent V, Gicquel B, et al. (2000) Variable human minisatellite-like regions in the Mycobacterium tuberculosis genome. Mol Microbiol 36: 762-771.

8. Beaumont MA, Rannala B (2004) The Bayesian revolution in genetics. Nat Rev Genet 5: 251-261.

9. Schlotterer C, Imhof M, Wang H, Nolte V, Harr B (2006) Low abundance of Escherichia coli microsatellites is associated with an extremely low mutation rate. J Evol Biol 19: 1671-1676.

10. Vogler AJ, Keys C, Nemoto Y, Colman RE, Jay Z, et al. (2006) Effect of repeat copy number on variable-number tandem repeat mutations in Escherichia coli O157:H7. J Bacteriol 188: 4253-4263.

11. Wierdl M, Dominska M, Petes TD (1997) Microsatellite instability in yeast: dependence on the length of the microsatellite. Genetics 146: 769-779.

12. Beaumont MA (1999) Detecting population expansion and decline using microsatellites. Genetics 153: 2013-2029.

13. Behar DM, Thomas MG, Skorecki K, Hammer MF, Bulygina E, et al. (2003) Multiple origins of Ashkenazi Levites: Y chromosome evidence for both Near Eastern and European ancestries. Am J Hum Genet 73: 768-779.

14. Storz JF, Beaumont MA (2002) Testing for genetic evidence of population expansion and contraction: an empirical analysis of microsatellite DNA variation using a hierarchical Bayesian model. Evolution Int J Org Evolution 56: 154-166.

15. Brosch R, Gordon SV, Marmiesse M, Brodin P, Buchrieser C, et al. (2002) A new evolutionary scenario for the Mycobacterium tuberculosis complex. Proc Natl Acad Sci U S A 99: 3684-3689.

16. Gagneux S, DeRiemer K, Van T, Kato-Maeda M, de Jong BC, et al. (2006) Variable host-pathogen compatibility in Mycobacterium tuberculosis. Proc Natl Acad Sci U S A 103: 2869-2873.

17. Huard RC, de Oliveira Lazzarini LC, Butler WR, van Soolingen D, Ho JL (2003) PCR-based method to differentiate the subspecies of the Mycobacterium tuberculosis complex on the basis of genomic deletions. J Clin Microbiol 41: 1637-1650.

18. Mostowy S, Cousins D, Brinkman J, Aranaz A, Behr MA (2002) Genomic deletions suggest a phylogeny for the Mycobacterium tuberculosis complex. J Infect Dis 186: 74-80.
