# Supplementary material for: Origin, Spread and Demography of the Mycobacterium tuberculosis Complex
Source: PLoS Pathog. 2008 Sep 26;4(9):e1000160. doi: 10.1371/journal.ppat.1000160 (PMC2528947; doi:10.1371/journal.ppat.1000160)
Supplement: Table S1 — List of the MTBC isolates used in this study. (0.08 MB DOC) [file ppat.1000160.s002.doc]

Isolate N° Species Origin Sample providers

10400/02 *M. africanum* Ghana Research Center Borstel, Germany Stefan Niemann

10438/01 *M. tuberculosis* Ghana Research Center Borstel, Germany Stefan Niemann

10439/01 *M. tuberculosis* Ghana Research Center Borstel, Germany Stefan Niemann

10445/01 *M. tuberculosis* Ghana Research Center Borstel, Germany Stefan Niemann

10446/01 *M. tuberculosis* Ghana Research Center Borstel, Germany Stefan Niemann

10458/02 *M. africanum* Ghana Research Center Borstel, Germany Stefan Niemann

10462/01 *M. africanum* Ghana Research Center Borstel, Germany Stefan Niemann

10469/01 *M. tuberculosis* Ghana Research Center Borstel, Germany Stefan Niemann

10470/01 *M. tuberculosis* Ghana Research Center Borstel, Germany Stefan Niemann

10473/01 *M. africanum* Ghana Research Center Borstel, Germany Stefan Niemann

10476/01 *M. africanum* Ghana Research Center Borstel, Germany Stefan Niemann

10480/01 *M. africanum* Ghana Research Center Borstel, Germany Stefan Niemann

10481/01 *M. tuberculosis* Ghana Research Center Borstel, Germany Stefan Niemann

10485/01 *M. africanum* Ghana Research Center Borstel, Germany Stefan Niemann

10486/01 *M. tuberculosis* Ghana Research Center Borstel, Germany Stefan Niemann

10493/01 *M. tuberculosis* Ghana Research Center Borstel, Germany Stefan Niemann

10494/01 *M. africanum* Ghana Research Center Borstel, Germany Stefan Niemann

10512/01 *M. africanum* Ghana Research Center Borstel, Germany Stefan Niemann

10514/01 *M. africanum* Ghana Research Center Borstel, Germany Stefan Niemann

10515/01 *M. tuberculosis* Ghana Research Center Borstel, Germany Stefan Niemann

10517/01 *M. africanum* Ghana Research Center Borstel, Germany Stefan Niemann

11443/99 *M. caprae* Germany Research Center Borstel, Germany Stefan Niemann

1290/03 *M. bovis* Ghana Research Center Borstel, Germany Stefan Niemann

1410/02 *M. africanum* Ghana Research Center Borstel, Germany Stefan Niemann

1417/02 *M. tuberculosis* Ghana Research Center Borstel, Germany Stefan Niemann

1428/02 *M. tuberculosis* Ghana Research Center Borstel, Germany Stefan Niemann

1438/02 *M. tuberculosis* Ghana Research Center Borstel, Germany Stefan Niemann

1443/02 *M. africanum* Ghana Research Center Borstel, Germany Stefan Niemann

1449/02 *M. africanum* Ghana Research Center Borstel, Germany Stefan Niemann

1465/02 *M. africanum* Ghana Research Center Borstel, Germany Stefan Niemann

1473/02 *M. africanum* Ghana Research Center Borstel, Germany Stefan Niemann

1479/00 *M. microti* Germany Research Center Borstel, Germany Stefan Niemann

1521/99 *M. tuberculosis* Uganda Research Center Borstel, Germany Stefan Niemann

1571/99 *M. tuberculosis* Uganda Research Center Borstel, Germany Stefan Niemann

1601/01 *M. bovis* Germany Research Center Borstel, Germany Stefan Niemann

1647/99 *M. tuberculosis* Uganda Research Center Borstel, Germany Stefan Niemann

1694/00 *M. caprae* Germany Research Center Borstel, Germany Stefan Niemann

1696/00 *M. caprae* Germany Research Center Borstel, Germany Stefan Niemann

1797/03 *M. tuberculosis* Germany Research Center Borstel, Germany Stefan Niemann

1805/02 *M. tuberculosis* Germany Research Center Borstel, Germany Stefan Niemann

2111/99 *M. tuberculosis* Uganda Research Center Borstel, Germany Stefan Niemann

2169/99 *M. tuberculosis* Uganda Research Center Borstel, Germany Stefan Niemann

2173/99 *M. tuberculosis* Uganda Research Center Borstel, Germany Stefan Niemann

2176/99 *M. tuberculosis* Uganda Research Center Borstel, Germany Stefan Niemann

2191/99 *M. tuberculosis* Uganda Research Center Borstel, Germany Stefan Niemann

2197/99 *M. tuberculosis* Uganda Research Center Borstel, Germany Stefan Niemann

2201/99 *M. tuberculosis* Uganda Research Center Borstel, Germany Stefan Niemann

2211/99 *M. tuberculosis* Uganda Research Center Borstel, Germany Stefan Niemann

2224/99 *M. tuberculosis* Uganda Research Center Borstel, Germany Stefan Niemann

2253/99 *M. tuberculosis* Uganda Research Center Borstel, Germany Stefan Niemann

2263/99 *M. tuberculosis* Uganda Research Center Borstel, Germany Stefan Niemann

2307/99 *M. tuberculosis* Uganda Research Center Borstel, Germany Stefan Niemann

2319/99 *M. tuberculosis* Uganda Research Center Borstel, Germany Stefan Niemann

2329/99 *M. tuberculosis* Uganda Research Center Borstel, Germany Stefan Niemann

2331/99 *M. tuberculosis* Uganda Research Center Borstel, Germany Stefan Niemann

2333/99 *M. tuberculosis* Uganda Research Center Borstel, Germany Stefan Niemann

2336/02 *M. tuberculosis* Germany Research Center Borstel, Germany Stefan Niemann

2379/99 *M. tuberculosis* Uganda Research Center Borstel, Germany Stefan Niemann

2569/02 *M. africanum* Ghana Research Center Borstel, Germany Stefan Niemann

2570/02 *M. tuberculosis* Ghana Research Center Borstel, Germany Stefan Niemann

2577/02 *M. africanum* Ghana Research Center Borstel, Germany Stefan Niemann

2582/02 *M. tuberculosis* Ghana Research Center Borstel, Germany Stefan Niemann

2597/02 *M. tuberculosis* Ghana Research Center Borstel, Germany Stefan Niemann

2637/02 *M. tuberculosis* Germany Research Center Borstel, Germany Stefan Niemann

287/99 *M. microti* Germany Research Center Borstel, Germany Stefan Niemann

3040/99 *M. prototuberculosis* Research Center Borstel, Germany Stefan Niemann

3041/99 *M. prototuberculosis* Research Center Borstel, Germany Stefan Niemann

3243/02 *M. tuberculosis* Kazakhstan Research Center Borstel, Germany Stefan Niemann

3256/02 *M. tuberculosis* Kazakhstan Research Center Borstel, Germany Stefan Niemann

3262/02 *M. tuberculosis* Kazakhstan Research Center Borstel, Germany Stefan Niemann

3277/02 *M. tuberculosis* Kazakhstan Research Center Borstel, Germany Stefan Niemann

3309/02 *M. tuberculosis* Kazakhstan Research Center Borstel, Germany Stefan Niemann

3310/02 *M. tuberculosis* Kazakhstan Research Center Borstel, Germany Stefan Niemann

3329/02 *M. tuberculosis* Kazakhstan Research Center Borstel, Germany Stefan Niemann

3342/02 *M. tuberculosis* Germany Research Center Borstel, Germany Stefan Niemann

3364/02 *M. tuberculosis* Kazakhstan Research Center Borstel, Germany Stefan Niemann

3482/03 *M. africanum* Ghana Research Center Borstel, Germany Stefan Niemann

4130/02 *M. tuberculosis* Germany Research Center Borstel, Germany Stefan Niemann

417/01 *M. microti* Germany Research Center Borstel, Germany Stefan Niemann

4217/02 *M. tuberculosis* Germany Research Center Borstel, Germany Stefan Niemann

4258/00 *M. bovis* Germany Research Center Borstel, Germany Stefan Niemann

4428/02 *M. tuberculosis* Kazakhstan Research Center Borstel, Germany Stefan Niemann

4431/02 *M. tuberculosis* Kazakhstan Research Center Borstel, Germany Stefan Niemann

4436/02 *M. tuberculosis* Kazakhstan Research Center Borstel, Germany Stefan Niemann

4445/02 *M. tuberculosis* Kazakhstan Research Center Borstel, Germany Stefan Niemann

4498/02 *M. tuberculosis* Kazakhstan Research Center Borstel, Germany Stefan Niemann

4499/02 *M. tuberculosis* Kazakhstan Research Center Borstel, Germany Stefan Niemann

4802/03 *M. africanum* Ghana Research Center Borstel, Germany Stefan Niemann

4804/03 *M. africanum* Ghana Research Center Borstel, Germany Stefan Niemann

4850/03 *M. tuberculosis* Germany Research Center Borstel, Germany Stefan Niemann

4993/02 *M. tuberculosis* Germany Research Center Borstel, Germany Stefan Niemann

5346/02 *M. bovis* Ghana Research Center Borstel, Germany Stefan Niemann

5357/02 *M. tuberculosis* Ghana Research Center Borstel, Germany Stefan Niemann

5383/02 *M. africanum* Ghana Research Center Borstel, Germany Stefan Niemann

5390/02 *M. tuberculosis* Ghana Research Center Borstel, Germany Stefan Niemann

5398/02 *M. africanum* Ghana Research Center Borstel, Germany Stefan Niemann

5400/02 *M. tuberculosis* Ghana Research Center Borstel, Germany Stefan Niemann

5429/02 *M. tuberculosis* Ghana Research Center Borstel, Germany Stefan Niemann

5432/02 *M. africanum* Ghana Research Center Borstel, Germany Stefan Niemann

5434/02 *M. africanum* Ghana Research Center Borstel, Germany Stefan Niemann

5468/02 *M. africanum* Ghana Research Center Borstel, Germany Stefan Niemann

5473/02 *M. africanum* Ghana Research Center Borstel, Germany Stefan Niemann

6427/01 *M. tuberculosis* Germany Research Center Borstel, Germany Stefan Niemann

6997/99 *M. microti* Germany Research Center Borstel, Germany Stefan Niemann

7011/02 *M. bovis* Germany Research Center Borstel, Germany Stefan Niemann

7072/01 *M. bovis* Germany Research Center Borstel, Germany Stefan Niemann

7190/03 *M. tuberculosis* Germany Research Center Borstel, Germany Stefan Niemann

7507/01 *M. tuberculosis* Germany Research Center Borstel, Germany Stefan Niemann

751/01 *M. bovis* Germany Research Center Borstel, Germany Stefan Niemann

7540/01 *M. bovis* Germany Research Center Borstel, Germany Stefan Niemann

7618/99 *M. caprae* Germany Research Center Borstel, Germany Stefan Niemann

7746/01 *M. tuberculosis* Germany Research Center Borstel, Germany Stefan Niemann

7747/01 *M. tuberculosis* Germany Research Center Borstel, Germany Stefan Niemann

7936/01 *M. tuberculosis* Germany Research Center Borstel, Germany Stefan Niemann

8163/02 *M. africanum* Ghana Research Center Borstel, Germany Stefan Niemann

8217/02 *M. bovis* Ghana Research Center Borstel, Germany Stefan Niemann

8236/02 *M. africanum* Ghana Research Center Borstel, Germany Stefan Niemann

8260/01 *M. tuberculosis* Germany Research Center Borstel, Germany Stefan Niemann

8303/02 *M. africanum* Ghana Research Center Borstel, Germany Stefan Niemann

8490/00 *M. bovis* Germany Research Center Borstel, Germany Stefan Niemann

8522/00 *M. caprae* Germany Research Center Borstel, Germany Stefan Niemann

8668/99 *M. microti* Germany Research Center Borstel, Germany Stefan Niemann

8753/00 *M. microti* Germany Research Center Borstel, Germany Stefan Niemann

8986/99 *M. caprae* Germany Research Center Borstel, Germany Stefan Niemann

9062/01 *M. caprae* Germany Research Center Borstel, Germany Stefan Niemann

9267/01 *M. tuberculosis* Germany Research Center Borstel, Germany Stefan Niemann

9398/01 *M. tuberculosis* Germany Research Center Borstel, Germany Stefan Niemann

9400/02 *M. tuberculosis* Germany Research Center Borstel, Germany Stefan Niemann

947/01 *M. tuberculosis* Germany Research Center Borstel, Germany Stefan Niemann

951/01 *M. bovis* Germany Research Center Borstel, Germany Stefan Niemann

9550/00 *M. africanum* ATTC Research Center Borstel, Germany Stefan Niemann

9564/00 *M. bovis* ATTC Research Center Borstel, Germany Stefan Niemann

9577/99 *M. caprae* Germany Research Center Borstel, Germany Stefan Niemann

9679/00 *M. tuberculosis* ATTC Research Center Borstel, Germany Stefan Niemann

9915/01 *M. tuberculosis* Germany Research Center Borstel, Germany Stefan Niemann

03BR0062 *M. tuberculosis* Belgium Institut Pasteur, Brussels, Caroline Allix, Maryse Fauville-Dufaux

03BR0162 *M. tuberculosis* Belgium Institut Pasteur, Brussels, Caroline Allix, Maryse Fauville-Dufaux

03BR0204 *M. tuberculosis* Belgium Institut Pasteur, Brussels, Caroline Allix, Maryse Fauville-Dufaux

03BR0139 *M. tuberculosis* Belgium Institut Pasteur, Brussels, Caroline Allix, Maryse Fauville-Dufaux

03BR0107 *M. tuberculosis* Belgium Institut Pasteur, Brussels, Caroline Allix, Maryse Fauville-Dufaux

04BR0159 *M. tuberculosis* Belgium Institut Pasteur, Brussels, Caroline Allix, Maryse Fauville-Dufaux

04BR0278 *M. tuberculosis* Belgium Institut Pasteur, Brussels, Caroline Allix, Maryse Fauville-Dufaux

04BR0032 *M. tuberculosis* Belgium Institut Pasteur, Brussels, Caroline Allix, Maryse Fauville-Dufaux

03BR0070 *M. tuberculosis* Belgium Institut Pasteur, Brussels, Caroline Allix, Maryse Fauville-Dufaux

02BR0024 *M. tuberculosis* Belgium Institut Pasteur, Brussels, Caroline Allix, Maryse Fauville-Dufaux

04BR0235 *M. tuberculosis* Belgium Institut Pasteur, Brussels, Caroline Allix, Maryse Fauville-Dufaux

03BR0027 *M. tuberculosis* Belgium Institut Pasteur, Brussels, Caroline Allix, Maryse Fauville-Dufaux

03BR0298 *M. tuberculosis* Belgium Institut Pasteur, Brussels, Caroline Allix, Maryse Fauville-Dufaux

03BR0116 *M. tuberculosis* Belgium Institut Pasteur, Brussels, Caroline Allix, Maryse Fauville-Dufaux

03BR0290 *M. tuberculosis* Belgium Institut Pasteur, Brussels, Caroline Allix, Maryse Fauville-Dufaux

03BR0109 *M. tuberculosis* Belgium Institut Pasteur, Brussels, Caroline Allix, Maryse Fauville-Dufaux

03BR0244 *M. tuberculosis* Belgium Institut Pasteur, Brussels, Caroline Allix, Maryse Fauville-Dufaux

02BR0046 *M. tuberculosis* Belgium Institut Pasteur, Brussels, Caroline Allix, Maryse Fauville-Dufaux

03BR0311 *M. tuberculosis* Belgium Institut Pasteur, Brussels, Caroline Allix, Maryse Fauville-Dufaux

03BR0041 *M. tuberculosis* Belgium Institut Pasteur, Brussels, Caroline Allix, Maryse Fauville-Dufaux

03BR0104 *M. tuberculosis* Belgium Institut Pasteur, Brussels, Caroline Allix, Maryse Fauville-Dufaux

02BR0061 *M. tuberculosis* Belgium Institut Pasteur, Brussels, Caroline Allix, Maryse Fauville-Dufaux

04BR0311 *M. tuberculosis* Belgium Institut Pasteur, Brussels, Caroline Allix, Maryse Fauville-Dufaux

04BR0142 *M. tuberculosis* Belgium Institut Pasteur, Brussels, Caroline Allix, Maryse Fauville-Dufaux

04BR0270 *M. tuberculosis* Belgium Institut Pasteur, Brussels, Caroline Allix, Maryse Fauville-Dufaux

03BR0145 *M. tuberculosis* Belgium Institut Pasteur, Brussels, Caroline Allix, Maryse Fauville-Dufaux

04BR0050 *M. tuberculosis* Belgium Institut Pasteur, Brussels, Caroline Allix, Maryse Fauville-Dufaux

04BR0309 *M. tuberculosis* Belgium Institut Pasteur, Brussels, Caroline Allix, Maryse Fauville-Dufaux

00-031 *M. tuberculosis* French Guyana Institut Pasteur, Guadeloupe, Christophe Sola, Nalin Rastogi

00-040 *M. tuberculosis* Guadeloupe Institut Pasteur, Guadeloupe, Christophe Sola, Nalin Rastogi

00-046 *M. tuberculosis* Guadeloupe Institut Pasteur, Guadeloupe, Christophe Sola, Nalin Rastogi

00-055 *M. tuberculosis* Guadeloupe Institut Pasteur, Guadeloupe, Christophe Sola, Nalin Rastogi

00-068 *M. tuberculosis* French Guyana Institut Pasteur, Guadeloupe, Christophe Sola, Nalin Rastogi

00-071 *M. tuberculosis* French Guyana Institut Pasteur, Guadeloupe, Christophe Sola, Nalin Rastogi

00-083 *M. tuberculosis* Guadeloupe Institut Pasteur, Guadeloupe, Christophe Sola, Nalin Rastogi

00-101 *M. tuberculosis* Martinique Institut Pasteur, Guadeloupe, Christophe Sola, Nalin Rastogi

12 *M. tuberculosis* Italy Institut Pasteur, Guadeloupe, Christophe Sola, Nalin Rastogi

13 *M. tuberculosis* Italy Institut Pasteur, Guadeloupe, Christophe Sola, Nalin Rastogi

21 *M. tuberculosis* Italy Institut Pasteur, Guadeloupe, Christophe Sola, Nalin Rastogi

34 *M. tuberculosis* Italy Institut Pasteur, Guadeloupe, Christophe Sola, Nalin Rastogi

40 *M. tuberculosis* Italy Institut Pasteur, Guadeloupe, Christophe Sola, Nalin Rastogi

41 *M. tuberculosis* Italy Institut Pasteur, Guadeloupe, Christophe Sola, Nalin Rastogi

43 *M. tuberculosis* Italy Institut Pasteur, Guadeloupe, Christophe Sola, Nalin Rastogi

53 *M. tuberculosis* Italy Institut Pasteur, Guadeloupe, Christophe Sola, Nalin Rastogi

69 *M. tuberculosis* Unknown Institut Pasteur, Guadeloupe, Christophe Sola, Nalin Rastogi

71 *M. tuberculosis* Italy Institut Pasteur, Guadeloupe, Christophe Sola, Nalin Rastogi

77 *M. tuberculosis* Unknown Institut Pasteur, Guadeloupe, Christophe Sola, Nalin Rastogi

80 *M. tuberculosis* Italy Institut Pasteur, Guadeloupe, Christophe Sola, Nalin Rastogi

83 *M. tuberculosis* Unknown Institut Pasteur, Guadeloupe, Christophe Sola, Nalin Rastogi

87 *M. tuberculosis* Italy Institut Pasteur, Guadeloupe, Christophe Sola, Nalin Rastogi

96 *M. tuberculosis* Unknown Institut Pasteur, Guadeloupe, Christophe Sola, Nalin Rastogi

101 *M. tuberculosis* Unknown Institut Pasteur, Guadeloupe, Christophe Sola, Nalin Rastogi

104 *M. tuberculosis* Italy Institut Pasteur, Guadeloupe, Christophe Sola, Nalin Rastogi

106 *M. tuberculosis* Italy Institut Pasteur, Guadeloupe, Christophe Sola, Nalin Rastogi

121 *M. tuberculosis* Unknown Institut Pasteur, Guadeloupe, Christophe Sola, Nalin Rastogi

131 *M. tuberculosis* Unknown Institut Pasteur, Guadeloupe, Christophe Sola, Nalin Rastogi

147 *M. tuberculosis* Italy Institut Pasteur, Guadeloupe, Christophe Sola, Nalin Rastogi

158 *M. tuberculosis* Italy Institut Pasteur, Guadeloupe, Christophe Sola, Nalin Rastogi

203 *M. tuberculosis* USA Institut Pasteur, Guadeloupe, Christophe Sola, Nalin Rastogi

217 *M. tuberculosis* USA Institut Pasteur, Guadeloupe, Christophe Sola, Nalin Rastogi

220 *M. tuberculosis* USA Institut Pasteur, Guadeloupe, Christophe Sola, Nalin Rastogi

559 *M. tuberculosis* Thailand Institut Pasteur, Guadeloupe, Christophe Sola, Nalin Rastogi

822 *M. tuberculosis* Thailand Institut Pasteur, Guadeloupe, Christophe Sola, Nalin Rastogi

94129 *M. tuberculosis* Guadeloupe Institut Pasteur, Guadeloupe, Christophe Sola, Nalin Rastogi

95054 *M. tuberculosis* Guadeloupe Institut Pasteur, Guadeloupe, Christophe Sola, Nalin Rastogi

96076 *M. tuberculosis* French Guyana Institut Pasteur, Guadeloupe, Christophe Sola, Nalin Rastogi

96085 *M. tuberculosis* Guadeloupe Institut Pasteur, Guadeloupe, Christophe Sola, Nalin Rastogi

96119 *M. tuberculosis* Guadeloupe Institut Pasteur, Guadeloupe, Christophe Sola, Nalin Rastogi

97010 *M. tuberculosis* Guadeloupe Institut Pasteur, Guadeloupe, Christophe Sola, Nalin Rastogi

97025 *M. tuberculosis* French Guyana Institut Pasteur, Guadeloupe, Christophe Sola, Nalin Rastogi

97062 *M. tuberculosis* Guadeloupe Institut Pasteur, Guadeloupe, Christophe Sola, Nalin Rastogi

97066 *M. tuberculosis* French Guyana Institut Pasteur, Guadeloupe, Christophe Sola, Nalin Rastogi

97070 *M. tuberculosis* French Guyana Institut Pasteur, Guadeloupe, Christophe Sola, Nalin Rastogi

97088 *M. tuberculosis* French Guyana Institut Pasteur, Guadeloupe, Christophe Sola, Nalin Rastogi

97108 *M. tuberculosis* French Guyana Institut Pasteur, Guadeloupe, Christophe Sola, Nalin Rastogi

97116 *M. tuberculosis* Guadeloupe Institut Pasteur, Guadeloupe, Christophe Sola, Nalin Rastogi

98004 *M. tuberculosis* French Guyana Institut Pasteur, Guadeloupe, Christophe Sola, Nalin Rastogi

98006 *M. tuberculosis* French Guyana Institut Pasteur, Guadeloupe, Christophe Sola, Nalin Rastogi

98008 *M. tuberculosis* French Guyana Institut Pasteur, Guadeloupe, Christophe Sola, Nalin Rastogi

98024 *M. tuberculosis* French Guyana Institut Pasteur, Guadeloupe, Christophe Sola, Nalin Rastogi

98026 *M. tuberculosis* Guadeloupe Institut Pasteur, Guadeloupe, Christophe Sola, Nalin Rastogi

98034 *M. tuberculosis* Guadeloupe Institut Pasteur, Guadeloupe, Christophe Sola, Nalin Rastogi

98036 *M. tuberculosis* French Guyana Institut Pasteur, Guadeloupe, Christophe Sola, Nalin Rastogi

98065 *M. tuberculosis* Guadeloupe Institut Pasteur, Guadeloupe, Christophe Sola, Nalin Rastogi

98077 *M. tuberculosis* Guadeloupe Institut Pasteur, Guadeloupe, Christophe Sola, Nalin Rastogi

98079 *M. tuberculosis* French Guyana Institut Pasteur, Guadeloupe, Christophe Sola, Nalin Rastogi

98094 *M. tuberculosis* French Guyana Institut Pasteur, Guadeloupe, Christophe Sola, Nalin Rastogi

98100 *M. tuberculosis* French Guyana Institut Pasteur, Guadeloupe, Christophe Sola, Nalin Rastogi

98104 *M. tuberculosis* French Guyana Institut Pasteur, Guadeloupe, Christophe Sola, Nalin Rastogi

98107 *M. tuberculosis* French Guyana Institut Pasteur, Guadeloupe, Christophe Sola, Nalin Rastogi

99005 *M. tuberculosis* French Guyana Institut Pasteur, Guadeloupe, Christophe Sola, Nalin Rastogi

99008 *M. tuberculosis* Guadeloupe Institut Pasteur, Guadeloupe, Christophe Sola, Nalin Rastogi

99016 *M. tuberculosis* Martinique Institut Pasteur, Guadeloupe, Christophe Sola, Nalin Rastogi

99019 *M. tuberculosis* French Guyana Institut Pasteur, Guadeloupe, Christophe Sola, Nalin Rastogi

99024 *M. tuberculosis* French Guyana Institut Pasteur, Guadeloupe, Christophe Sola, Nalin Rastogi

99035 *M. tuberculosis* Guadeloupe Institut Pasteur, Guadeloupe, Christophe Sola, Nalin Rastogi

99043 *M. tuberculosis* Guadeloupe Institut Pasteur, Guadeloupe, Christophe Sola, Nalin Rastogi

99060 *M. tuberculosis* French Guyana Institut Pasteur, Guadeloupe, Christophe Sola, Nalin Rastogi

99102 *M. tuberculosis* Martinique Institut Pasteur, Guadeloupe, Christophe Sola, Nalin Rastogi

99120 *M. tuberculosis* Guadeloupe Institut Pasteur, Guadeloupe, Christophe Sola, Nalin Rastogi

99123 *M. tuberculosis* Guadeloupe Institut Pasteur, Guadeloupe, Christophe Sola, Nalin Rastogi

B2 *M. tuberculosis* Barbados Institut Pasteur, Guadeloupe, Christophe Sola, Nalin Rastogi

B4 *M. tuberculosis* Barbados Institut Pasteur, Guadeloupe, Christophe Sola, Nalin Rastogi

bov01 *M. bovis* Guadeloupe Institut Pasteur, Guadeloupe, Christophe Sola, Nalin Rastogi

bov929 *M. bovis* Unknown Institut Pasteur, Guadeloupe, Christophe Sola, Nalin Rastogi

IPC32 *M. tuberculosis* French Guyana Institut Pasteur, Guadeloupe, Christophe Sola, Nalin Rastogi

IPC35 *M. tuberculosis* French Guyana Institut Pasteur, Guadeloupe, Christophe Sola, Nalin Rastogi

IPC47 *M. tuberculosis* French Guyana Institut Pasteur, Guadeloupe, Christophe Sola, Nalin Rastogi

M28 *M. tuberculosis* Italy Institut Pasteur, Guadeloupe, Christophe Sola, Nalin Rastogi

M6 *M. tuberculosis* Martinique Institut Pasteur, Guadeloupe, Christophe Sola, Nalin Rastogi

Mt14323 *M. tuberculosis* The Netherlands Institut Pasteur, Guadeloupe, Christophe Sola, Nalin Rastogi

1 *M. tuberculosis* The Netherlands RIVM, Bilthoven, the Netherlands Kristin Kremer, Dick Van Soolingen

2 *M. bovis BCG* The Netherlands RIVM, Bilthoven, the Netherlands Kristin Kremer, Dick Van Soolingen

4 *M. tuberculosis* Rwanda RIVM, Bilthoven, the Netherlands Kristin Kremer, Dick Van Soolingen

6 *M. bovis*  The Netherlands RIVM, Bilthoven, the Netherlands Kristin Kremer, Dick Van Soolingen

7 *M. tuberculosis* Mongolia RIVM, Bilthoven, the Netherlands Kristin Kremer, Dick Van Soolingen

8 *M. tuberculosis* Vietnam RIVM, Bilthoven, the Netherlands Kristin Kremer, Dick Van Soolingen

10 *M. tuberculosis* Equador RIVM, Bilthoven, the Netherlands Kristin Kremer, Dick Van Soolingen

13 *M. tuberculosis* Sri Lanka RIVM, Bilthoven, the Netherlands Kristin Kremer, Dick Van Soolingen

14 *M. tuberculosis* South Africa RIVM, Bilthoven, the Netherlands Kristin Kremer, Dick Van Soolingen

18 *M. tuberculosis* USA RIVM, Bilthoven, the Netherlands Kristin Kremer, Dick Van Soolingen

19 *M. tuberculosis* India RIVM, Bilthoven, the Netherlands Kristin Kremer, Dick Van Soolingen

20 *M. tuberculosis* Mongolia RIVM, Bilthoven, the Netherlands Kristin Kremer, Dick Van Soolingen

21 *M. tuberculosis* Zimbabwe RIVM, Bilthoven, the Netherlands Kristin Kremer, Dick Van Soolingen

23 *M. tuberculosis* Honduras RIVM, Bilthoven, the Netherlands Kristin Kremer, Dick Van Soolingen

24 *M. bovis* Saoudi Arabia RIVM, Bilthoven, the Netherlands Kristin Kremer, Dick Van Soolingen

25 *M. microti* UK RIVM, Bilthoven, the Netherlands Kristin Kremer, Dick Van Soolingen

28 *M. tuberculosis* Sri Lanka RIVM, Bilthoven, the Netherlands Kristin Kremer, Dick Van Soolingen

29 *M. tuberculosis* India RIVM, Bilthoven, the Netherlands Kristin Kremer, Dick Van Soolingen

30 *M. tuberculosis* South Africa RIVM, Bilthoven, the Netherlands Kristin Kremer, Dick Van Soolingen

32 *M. tuberculosis* Russia RIVM, Bilthoven, the Netherlands Kristin Kremer, Dick Van Soolingen

33 *M. tuberculosis* The Netherlands RIVM, Bilthoven, the Netherlands Kristin Kremer, Dick Van Soolingen

34 *M. tuberculosis* Malaysia RIVM, Bilthoven, the Netherlands Kristin Kremer, Dick Van Soolingen

35 *M. tuberculosis* Rwanda RIVM, Bilthoven, the Netherlands Kristin Kremer, Dick Van Soolingen

36 *M. tuberculosis* India RIVM, Bilthoven, the Netherlands Kristin Kremer, Dick Van Soolingen

37 *M. tuberculosis* Uganda RIVM, Bilthoven, the Netherlands Kristin Kremer, Dick Van Soolingen

40 *M. tuberculosis* Burundi RIVM, Bilthoven, the Netherlands Kristin Kremer, Dick Van Soolingen

41 *M. tuberculosis* Chili RIVM, Bilthoven, the Netherlands Kristin Kremer, Dick Van Soolingen

42 *M. tuberculosis* Tahiti RIVM, Bilthoven, the Netherlands Kristin Kremer, Dick Van Soolingen

43 *M. tuberculosis* China RIVM, Bilthoven, the Netherlands Kristin Kremer, Dick Van Soolingen

44 *M. tuberculosis* Thailand RIVM, Bilthoven, the Netherlands Kristin Kremer, Dick Van Soolingen

45 *M. tuberculosis* Malaysia RIVM, Bilthoven, the Netherlands Kristin Kremer, Dick Van Soolingen

46 *M. tuberculosis* Chili RIVM, Bilthoven, the Netherlands Kristin Kremer, Dick Van Soolingen

47 *M. bovis*  The Netherlands RIVM, Bilthoven, the Netherlands Kristin Kremer, Dick Van Soolingen

49 *M. tuberculosis* Tanzania RIVM, Bilthoven, the Netherlands Kristin Kremer, Dick Van Soolingen

50 *M. tuberculosis* Czech Republic RIVM, Bilthoven, the Netherlands Kristin Kremer, Dick Van Soolingen

51 *M. tuberculosis* The Netherlands RIVM, Bilthoven, the Netherlands Kristin Kremer, Dick Van Soolingen

53 *M. tuberculosis* Argentina RIVM, Bilthoven, the Netherlands Kristin Kremer, Dick Van Soolingen

54 *M. tuberculosis* Thailand RIVM, Bilthoven, the Netherlands Kristin Kremer, Dick Van Soolingen

58 *M. tuberculosis* Argentina RIVM, Bilthoven, the Netherlands Kristin Kremer, Dick Van Soolingen

60 *M. tuberculosis* Bolivia RIVM, Bilthoven, the Netherlands Kristin Kremer, Dick Van Soolingen

62 *M. microti* UK RIVM, Bilthoven, the Netherlands Kristin Kremer, Dick Van Soolingen

63 *M. tuberculosis* Italy RIVM, Bilthoven, the Netherlands Kristin Kremer, Dick Van Soolingen

65 *M. tuberculosis* The Netherlands RIVM, Bilthoven, the Netherlands Kristin Kremer, Dick Van Soolingen

66 *M. tuberculosis* Spain RIVM, Bilthoven, the Netherlands Kristin Kremer, Dick Van Soolingen

67 *M. tuberculosis* Comoro Islands RIVM, Bilthoven, the Netherlands Kristin Kremer, Dick Van Soolingen

69 *M. bovis*  The Netherlands RIVM, Bilthoven, the Netherlands Kristin Kremer, Dick Van Soolingen

71 *M. bovis BCG* Japan RIVM, Bilthoven, the Netherlands Kristin Kremer, Dick Van Soolingen

73 *M. bovis* The Netherlands RIVM, Bilthoven, the Netherlands Kristin Kremer, Dick Van Soolingen

74 *M. tuberculosis* India RIVM, Bilthoven, the Netherlands Kristin Kremer, Dick Van Soolingen

76 *M. bovis*  Argentina RIVM, Bilthoven, the Netherlands Kristin Kremer, Dick Van Soolingen

77 *M. tuberculosis* Tunisia RIVM, Bilthoven, the Netherlands Kristin Kremer, Dick Van Soolingen

81 *M. bovis*  Argentina RIVM, Bilthoven, the Netherlands Kristin Kremer, Dick Van Soolingen

82 *M. tuberculosis* Canada RIVM, Bilthoven, the Netherlands Kristin Kremer, Dick Van Soolingen

83 *M. bovis BCG* Russia RIVM, Bilthoven, the Netherlands Kristin Kremer, Dick Van Soolingen

85 *M. bovis*  The Netherlands RIVM, Bilthoven, the Netherlands Kristin Kremer, Dick Van Soolingen

86 *M. tuberculosis* Bolivia RIVM, Bilthoven, the Netherlands Kristin Kremer, Dick Van Soolingen

87 *M. tuberculosis* USA RIVM, Bilthoven, the Netherlands Kristin Kremer, Dick Van Soolingen

90 *M. tuberculosis* South Korea RIVM, Bilthoven, the Netherlands Kristin Kremer, Dick Van Soolingen

93 *M. tuberculosis* Tanzania RIVM, Bilthoven, the Netherlands Kristin Kremer, Dick Van Soolingen

95 *M. tuberculosis* Spain RIVM, Bilthoven, the Netherlands Kristin Kremer, Dick Van Soolingen

97 *M. tuberculosis* Uganda RIVM, Bilthoven, the Netherlands Kristin Kremer, Dick Van Soolingen

98 *M. tuberculosis* Equador RIVM, Bilthoven, the Netherlands Kristin Kremer, Dick Van Soolingen

101 *M. bovis*  Argentina RIVM, Bilthoven, the Netherlands Kristin Kremer, Dick Van Soolingen

109 *M. tuberculosis* USA RIVM, Bilthoven, the Netherlands Kristin Kremer, Dick Van Soolingen

111 *M. tuberculosis* South Korea RIVM, Bilthoven, the Netherlands Kristin Kremer, Dick Van Soolingen

112 *M. tuberculosis* The Netherlands RIVM, Bilthoven, the Netherlands Kristin Kremer, Dick Van Soolingen

116 *M. prototuberculosis* Somalia RIVM, Bilthoven, the Netherlands Kristin Kremer, Dick Van Soolingen

117 *M. bovis*  Argentina RIVM, Bilthoven, the Netherlands Kristin Kremer, Dick Van Soolingen

118 *M. tuberculosis* Honduras RIVM, Bilthoven, the Netherlands Kristin Kremer, Dick Van Soolingen

120 *M. tuberculosis* Burundi RIVM, Bilthoven, the Netherlands Kristin Kremer, Dick Van Soolingen

121 *M. tuberculosis* CAR RIVM, Bilthoven, the Netherlands Kristin Kremer, Dick Van Soolingen

123 *M. tuberculosis* Czech Republic RIVM, Bilthoven, the Netherlands Kristin Kremer, Dick Van Soolingen

126 *M. bovis*  Argentina RIVM, Bilthoven, the Netherlands Kristin Kremer, Dick Van Soolingen

130 *M. bovis*  The Netherlands RIVM, Bilthoven, the Netherlands Kristin Kremer, Dick Van Soolingen

574 *M. prototuberculosis* Djibouti Institut Pasteur, Paris, Maria-Cristina Gutierrez

970130 *M. prototuberculosis* Switzerland Institut Pasteur, Paris, Maria-Cristina Gutierrez

981514 *M. prototuberculosis* Djibouti Institut Pasteur, Paris, Maria-Cristina Gutierrez

990121 *M. prototuberculosis* France Institut Pasteur, Paris, Maria-Cristina Gutierrez

990160 *M. prototuberculosis* Djibouti Institut Pasteur, Paris, Maria-Cristina Gutierrez

990161 *M. prototuberculosis* France Institut Pasteur, Paris, Maria-Cristina Gutierrez

990263 *M. prototuberculosis* France Institut Pasteur, Paris, Maria-Cristina Gutierrez

990264 *M. prototuberculosis* Djibouti Institut Pasteur, Paris, Maria-Cristina Gutierrez

19980862 *M. prototuberculosis* Djibouti Institut Pasteur, Paris, Maria-Cristina Gutierrez

19980863 *M. prototuberculosis* Djibouti Institut Pasteur, Paris, Maria-Cristina Gutierrez

19980864 *M. prototuberculosis* Djibouti Institut Pasteur, Paris, Maria-Cristina Gutierrez

19990516 *M. prototuberculosis* Djibouti Institut Pasteur, Paris, Maria-Cristina Gutierrez

19990589 *M. prototuberculosis* Djibouti Institut Pasteur, Paris, Maria-Cristina Gutierrez

19990711 *M. prototuberculosis* Djibouti Institut Pasteur, Paris, Maria-Cristina Gutierrez

19991704 *M. prototuberculosis* Djibouti Institut Pasteur, Paris, Maria-Cristina Gutierrez

19991705 *M. prototuberculosis* Djibouti Institut Pasteur, Paris, Maria-Cristina Gutierrez

19991708 *M. prototuberculosis* Djibouti Institut Pasteur, Paris, Maria-Cristina Gutierrez

19991709 *M. prototuberculosis* Djibouti Institut Pasteur, Paris, Maria-Cristina Gutierrez

20000342 *M. prototuberculosis* Djibouti Institut Pasteur, Paris, Maria-Cristina Gutierrez

20000473 *M. prototuberculosis* Djibouti Institut Pasteur, Paris, Maria-Cristina Gutierrez

20000587 *M. prototuberculosis* Djibouti Institut Pasteur, Paris, Maria-Cristina Gutierrez

20001049 *M. prototuberculosis* France Institut Pasteur, Paris, Maria-Cristina Gutierrez

20001155 *M. prototuberculosis* Djibouti Institut Pasteur, Paris, Maria-Cristina Gutierrez

20001245 *M. prototuberculosis* France Institut Pasteur, Paris, Maria-Cristina Gutierrez

20001246 *M. prototuberculosis* Djibouti Institut Pasteur, Paris, Maria-Cristina Gutierrez

20001247 *M. prototuberculosis* Djibouti Institut Pasteur, Paris, Maria-Cristina Gutierrez

20001248 *M. prototuberculosis* Djibouti Institut Pasteur, Paris, Maria-Cristina Gutierrez

20010188 *M. prototuberculosis* Djibouti Institut Pasteur, Paris, Maria-Cristina Gutierrez

20010389 *M. prototuberculosis* Djibouti Institut Pasteur, Paris, Maria-Cristina Gutierrez

20010933 *M. prototuberculosis* Djibouti Institut Pasteur, Paris, Maria-Cristina Gutierrez

140010059 *M. prototuberculosis* Tahiti Institut Pasteur, Paris, Maria-Cristina Gutierrez

140010060 *M. prototuberculosis* France Institut Pasteur, Paris, Maria-Cristina Gutierrez

140010061 *M. prototuberculosis* France Institut Pasteur, Paris, Maria-Cristina Gutierrez

200110390 *M. prototuberculosis* Djibouti Institut Pasteur, Paris, Maria-Cristina Gutierrez

19990515B *M. prototuberculosis* Djibouti Institut Pasteur, Paris, Maria-Cristina Gutierrez
