# Supplementary material for: Origin, Spread and Demography of the Mycobacterium tuberculosis Complex
Source: PLoS Pathog. 2008 Sep 26;4(9):e1000160. doi: 10.1371/journal.ppat.1000160 (PMC2528947; doi:10.1371/journal.ppat.1000160)
Supplement: Figure S1 — Bubble-graph representation of allele frequencies for the different MIRU loci. Allele size (number of repeats) on the y-axis, and source populations on the x-axis. (2.07 MB PDF) [file ppat.1000160.s003.pdf]

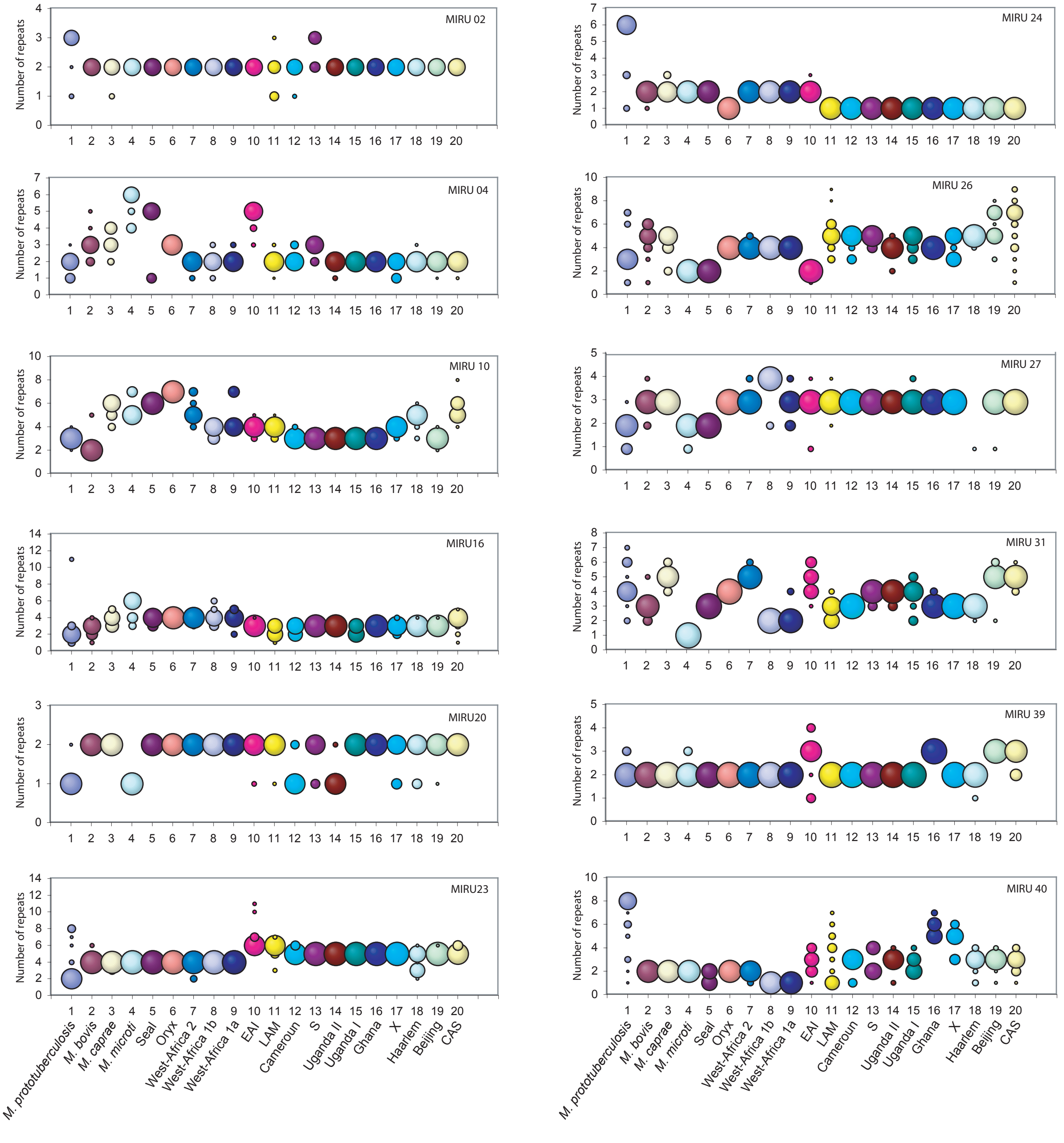
MIRU 02
MIRU 24
MIRU 04
MIRU 26
MIRU 10
MIRU 27
MIRU16
MIRU 31
MIRU20
MIRU 39
MIRU23
MIRU 40
Number of repeats
M. prototuberculosis
M. bovis
M. caprae
M. microti
Seal
Oryx
West-Africa 2
West-Africa 1b
West-Africa 1a
EAI
LAM
Cameroun
S
Uganda II
Uganda I
Ghana
X
Haarlem
Beijing
CAS

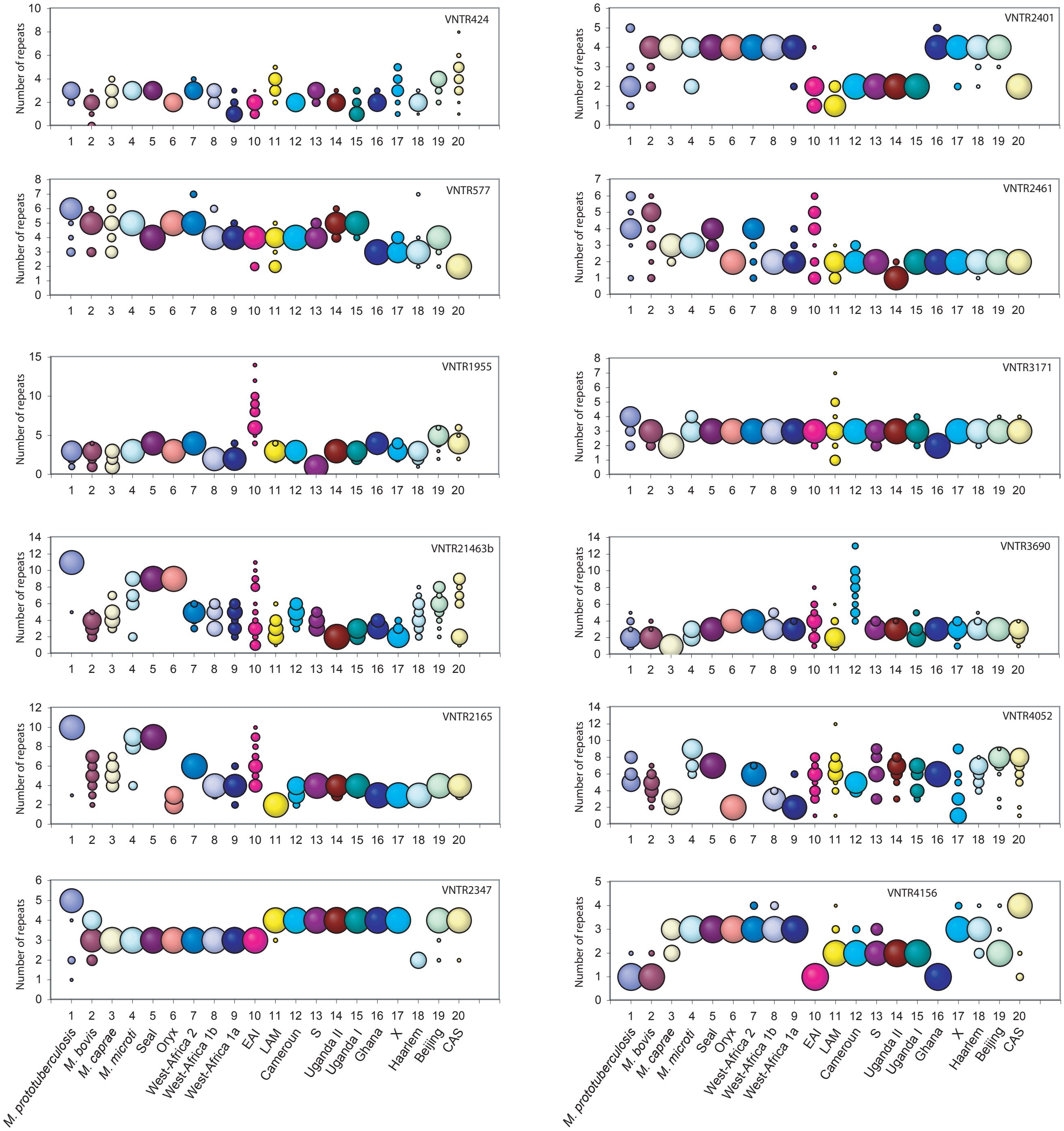

VNTR424
VNTR2401
VNTR577
VNTR2461
VNTR1955
VNTR3171
VNTR21463b
VNTR3690
VNTR2165
VNTR4052
VNTR2347
VNTR4156
Number of repeats
M. prototuberculosis
M. bovis
M. caprae
M. microti
Seal
Oryx
West-Africa 2
West-Africa 1b
West-Africa 1a
EAI
LAM
Cameroun
S
Uganda II
Uganda I
Ghana
X
Haarlem
Beijing
CAS
